# A Systematic Review of Social Presence: Definition, Antecedents, and Implications

**DOI:** 10.3389/frobt.2018.00114

**Published:** 2018-10-15

**Authors:** Catherine S. Oh, Jeremy N. Bailenson, Gregory F. Welch

**Affiliations:** ^1^Virtual Human Interaction Lab, Department of Communication, Stanford University, Stanford, CA, United States; ^2^College of Nursing, Department of Computer Science, Institute for Simulation & Training (Synthetic Reality Lab), University of Central Florida, Orlando, FL, United States

**Keywords:** social presence, presence, virtual reality, virtual environments, immersion, computer-mediated communication

## Abstract

Social presence, or the feeling of being there with a “real” person, is a crucial component of interactions that take place in virtual reality. This paper reviews the concept, antecedents, and implications of social presence, with a focus on the literature regarding the predictors of social presence. The article begins by exploring the concept of social presence, distinguishing it from two other dimensions of presence—telepresence and self-presence. After establishing the definition of social presence, the article offers a systematic review of 233 separate findings identified from 152 studies that investigate the factors (i.e., immersive qualities, contextual differences, and individual psychological traits) that predict social presence. Finally, the paper discusses the implications of heightened social presence and when it does and does not enhance one's experience in a virtual environment.

## Introduction

Since its conceptualization, virtual reality (VR) has been touted as a novel communication medium that would radically change the way people interact with each other (Biocca and Levy, 2013). William Gibson famously described cyberspace as a “consensual hallucination experienced daily by billions of legitimate operators, in every nation” (Gibson, 1984, p. 51), portraying the social nature of “stepping through a barrier” (Slater and Wilbur, 1997, p. 2) into the virtual environment. More recently, VR pioneer Jaron Lanier, expressed his hope that VR would lead to new and exciting forms of communication (Lanier, 2017).

Despite the conceptualization of VR as a social medium wherein individuals could co-exist and interact with each other (Biocca and Levy, 2013), much of the early research on VR technology focused on single-user head-mounted display (HMD) systems that typically were not available outside of the laboratory. In more recent years, however, VR technology has rapidly made its transition from lab to home in various forms. This increased accessibility of VR technology has fueled a renewed interest in the social applications of VR, which is exemplified by the launch of multiple platforms including AltSpace VR, Facebook Spaces, High Fidelity, Normal VR, Oculus Medium, Rec Room, Sansar, and VR Chat.

One of the primary attractions of VR is purported to be the level of *social presence* it affords in comparison to other forms of technology-mediated communication. Social presence refers to the subjective experience of being present with a “real” person and having access to his or her thoughts and emotions (Biocca, 1997); as such, one of the primary goals of networked communication systems is to offer higher levels of social presence (Biocca and Harms, 2002). Earlier forms of text-based computer-mediated communication (CMC) offered a limited amount of verbal and nonverbal information, which subsequently reduced the level of social presence people could feel within a set amount of time. Recent advancements in technology, however, have made media far more immersive than the past; in contrast to earlier forms of CMC, wherein individuals could only use text-based cues to express themselves, VR systems have the capacity to offer a wide array of social cues through visual, audio, haptic, and—to a lesser extent—olfactory information. It is therefore necessary to understand how different technological features influence perceptions of social presence to inform the design of VR platforms.

Researchers have also found that social presence can be influenced by contextual and individual factors that impact perceptions of the psychological distance between interactants (e.g., Siriaraya and Ang, 2012; Kang and Gratch, 2014; Verhagen et al., 2014). Studies conducted by these researchers show that the communication context as well as the individual traits of the interactants can influence perceptions of social presence. One of the most significant contributions of this line of research is that it sheds light on when increasing immersion is (and is not) necessary in order to induce stronger feelings of social presence. In a similar vein, these studies can inform both academic and applied researchers on how to maximize the amount of social presence one can feel within a given virtual environment.

To understand the concept, antecedents, and implications of social presence, we will first define two key concepts of the current paper, namely immersion and presence. Then we will offer a brief description of two separate dimensions of presence—telepresence and self-presence—to distinguish them from social presence. The remainder of the paper will focus on synthesizing the research on the antecedents of social presence to explore what does (and does not) impact perceptions of social presence.

### Immersion and the dimensions of presence

While some researchers use the terms “immersion” and “presence” interchangeably, distinguishing the two concepts allows for a better understanding of the difference between the *technological qualities* and *psychological experiences* afforded by mediated communication. Immersion can be defined as a medium's technological capacity to generate realistic experiences that can remove people from their physical reality (Slater and Wilbur, 1997). When defined in this way, immersion can be objectively measured by the technological affordances of a medium. Media are more immersive when they can deliver “an inclusive, extensive, surrounding and vivid illusion of reality to the senses of a human participant” (Slater and Wilbur, 1997, p. 604). Features such as audio and visual quality, frame rate, stereoscopy, and field of view can impact the extent to which a system is immersive (Welch et al., 1996; Johnson and Stewart, 1999; Skalski and Whitbred, 2010; Cummings and Bailenson, 2016).

In contrast to immersion, presence is the subjective experience of actually being in the mediated virtual environment (Slater and Wilbur, 1997; Witmer and Singer, 1998; Walther and Parks, 2002). As presence is needed for people to fully experience a virtual environment, it has been the focus of both applied and academic work on virtual reality (Cummings et al., 2012). Presence can be further divided into three distinct subcategories: telepresence (spatial presence), self-presence, and social presence (Lee, 2004).

Telepresence can be defined as “the extent to which one feels present in the mediated environment, rather than in the immediate physical environment” (Steuer, 1992, p. 75). This dimension of presence relates strongly to how vividly the user experiences the environmental and spatial properties of the mediated environment. When the perception of telepresence is strong, people should no longer be aware that their experiences are being mediated through technology (Lombard and Ditton, 1997).

In contrast to telepresence, self-presence is the extent to which the “virtual self is experienced as the actual self” (Aymerich-Franch et al., 2012, p. 1). This dimension of presence differs from telepresence, as it is not related to how vividly one experiences his or her surroundings, but rather, how connected one feels to his or her virtual body, emotions, or identity (Ratan and Hasler, 2009).

Finally, social presence or co-presence, refers to the “sense of being with another” (Biocca et al., 2003, p. 456) and is dependent on the ease with which one perceives to have “the access to the intelligence, intentions, and sensory impressions of another” (Biocca, 1997, p. 22). The concept was first introduced as a theoretical framework to understand the interactions that took place on different forms of media (Short et al., 1976). Social presence differs from both telepresence and self-presence, as it requires a co-present entity that appears to be sentient. Social presence is an integral part of virtual environments that mediate people; without social presence, the mediated other is merely experienced as an artificial entity and not a social being (Lee et al., 2006a).

### The evolution of social presence

Social presence was first conceptualized by Short et al. (1976) and was defined as the salience of the interactants and their interpersonal relationship during a mediated conversation. According to Short et al. (1976), intimacy and immediacy are the two core components of social presence. These two concepts are closely related to each other; intimacy refers to the feeling of connectedness that communicators feel during an interaction, while immediacy is the psychological distance between the communicators. Both intimacy and immediacy are determined by verbal and nonverbal cues such as facial expressions, vocal cues, gestures, and physical appearance (Gunawardena and Zittle, 1997). Short and colleagues argued that some media were more capable at delivering these cues, while others were not, emphasizing that social presence was a “quality of the medium itself” (Short et al., 1976, p. 65).

The view that social presence is technologically determined was also echoed by early CMC researchers who endorsed the cues-filtered-out perspective (see Walther and Parks, 2002 for review). For example, media richness theory (Daft and Lengel, 1986) claimed that different media varied in their ability to reproduce “rich” social information (e.g., immediate feedback, language variety, personalization, number of cues), thereby making some media more appropriate than others for certain tasks. Put otherwise, certain media are inherently superior to others in achieving a specific communication goal. While some researchers have since rejected this technology-driven conceptualization of social presence (e.g., Walther, 1992), others continue to examine whether people feel different levels of social presence when interacting via a specific medium compared to another. Studies that focus on how the modality or specific technological affordances of a medium (e.g., immersive features) impact social presence are based on the assumption that certain affordances of a medium can increase or decrease social presence when all other circumstances are equal (e.g., Axelsson et al., 2001; Moreno and Mayer, 2004; Zhan and Mei, 2013).

In contrast to these medium-centric views of social presence, Walther (1992) argued that individuals are capable of adapting to different communication media, and can thus achieve their communication goals accordingly. From this perspective, the experience of social presence is highly contingent on the interactants, rather than the medium itself. This view is known as social information processing theory (SIPT). According to this theory, communication environments that offer fewer verbal and/or nonverbal cues (e.g., text-based CMC) can produce equal levels of intimacy as face-to-face (FtF) communication, although it may take more time. Walther (1996) later expanded this theory to posit that people who communicate via text-based CMC platforms could, in some cases, achieve even higher levels of social presence than FtF interactants by carefully selecting which facets of themselves they wish to reveal (i.e., hyperpersonal model of communication). Subsequent studies have since shown that individuals adopt different strategies to convey socioemotional cues on platforms with relatively limited verbal and nonverbal cues (e.g., Ramirez et al., 2002; Antheunis et al., 2010).

While both SIPT and the hyperpersonal model posit that technology does not solely determine the level of social presence a medium can afford, it is important to note that neither perspective denies the inherent differences between media. When individuals are only given limited communication options (e.g., short timespan, specific task type, etc.), it is probable that the technological features of the environment will influence the level of social presence a person feels. At the same time, however, this perspective offers a more nuanced view of social presence; while the immersive qualities (i.e., computer system's technological capacity to deliver a vivid experience) can impact social presence, individual communication strategies as well as contextual differences have a significant effect on social presence.

### Why does social presence matter?

While both telepresence and self-presence have received academic focus, social presence has been considered to be particularly important within virtual environments with social actors (regardless of whether they are controlled by actual people or computer algorithms). This is due to the impact of social presence on social influence. Studies have shown that social presence is associated with a variety of positive communication outcomes, such as persuasion and attraction (e.g., Fogg and Tseng, 1999; Lee et al., 2006a). For example, Hassanein and Head (2007) found that social presence was positively associated with trust, enjoyment, and perceived usefulness of an online shopping website, which led to greater purchase intentions. Another study wherein social presence was operationalized to focus on the extent to which participants felt like they were together with their partner similarly found that social presence predicted attraction toward a physically embodied agent (i.e., robot; Lee et al., 2006a).

### Antecedents of social presence

Because social presence often predicts positive communication outcomes, both academic researchers and practitioners have displayed a great interest in studying the factors that increase social presence. By reviewing 233 separate findings identified from 152 studies, we found that researchers have most often explored the influence of immersive qualities, contextual differences, and individual psychological traits on social presence (see Table 1). However, to the best of our knowledge, little effort has been made to synthesize the findings of these studies (for an exception, see Cummings and Wertz, 2018). Consequently, it is difficult to have a holistic understanding of which features are the most influential in predicting social presence. This paper attempts to overcome this shortcoming by offering a systematic review of the extant literature on the immersive, contextual, and psychological features that impact perceived social presence. The results, details, and general information of the studies that were reviewed are available in Tables 1–3.

**Table 1:** Summary of study results.

**Predictor**	**References**	**Details**	**Outcome**	**Notes**
**IMMERSION**				
Modality	Appel et al., 2012	Text vs. Avatar	+	
	Alge et al., 2003	CMC vs. FtF	+	
	Alghamdi et al., 2016 (Study 2)	Desktop vs. HMD	Null	
	Axelsson et al., 2001	Desktop vs. CAVE	Null	
	Bailenson et al., 2006	Audio vs. Audio+Video vs. Audio+Emotibox	Others > Audio+Emotibox	
	Bente et al., 2008	Text vs. Audio vs. Audio+Video vs. Audio+Avatar	Others > Text	
	Cortese and Seo, 2012	CMC vs. FTF	+	
	de Greef, 2014	Audio vs. Audio+Video	+*	*Moderated by gender
	de Greef and Ijsselsteijn, 2001	Audio vs. Audio+Video	+	
	Francescato et al., 2006	CMC vs. FtF	Null	
	Gimpel et al., 2016	Text vs. Audio vs. Audio+Video	+	
	Hauber et al., 2005	2D vs. 3D vs. FtF	Others < FtF	
	Hauber et al., 2006	2D vs. 3D-local vs. 3D-remote vs. FtF	Others < FtF	
	Hauber et al., 2012	Video vs. Video-CVE vs. Stereo large-screen video-CVE vs. FTF	Others < FtF*	*Moderated by gender
	Heldal et al., 2005	IPT*-IPT vs. IPT-HMD vs. IPT-Desktop vs. Desktop-Desktop	IPT-IPT/HMD >Desktop-Desktop/IPT	*IPT: Immersive Projection Technology
	Hills, 2005 (Study 1)	2D vs. 3D vs. FtF	+	
	Hills, 2005 (Study 2)	2D vs. 3D	+	
	Homer et al., 2008	Audio vs. Audio+Video	Null	
	Järvelä et al., 2016	Nonverbal vs. Verbal	+*	*Moderated by physical proximity
	Jin, 2009	Text vs. Audio	–*	*Moderated by product involvement
	Johnsen and Lok, 2008	Large screen display vs. HMD	Null	
	Jung et al., 2017	Picture vs. Video	+	
	Kim et al., 2014	Text vs. Text+Video	+	
	Kim et al., 2013b	Text vs. Audio	+	
	Kothgassner et al., 2014	HMD vs. FtF	+	
	Lee, 2013	Television vs. Twitter	–*	*Moderated by Need-for-Cognition
	Lee and Jang, 2013 (Study 1)	Newspaper vs. Twitter	+^*^	*Moderated by affiliative tendency
	Lee and Jang, 2013 (Study 2)	Newspaper vs. Twitter	+^*^	*Moderated by affiliative tendency
	Lee and Shin, 2014	Newspaper vs. Twitter	+^*^	*Moderated by transportability
	Moreno and Mayer, 2004	Desktop vs. HMD	Null	
	Nam et al., 2008	Visual+Haptic vs. Visual+Haptic+Audio	+	
	Nowak et al., 2009	Text vs. Video	+	
	Qiu and Benbasat, 2005	Text vs. Audio vs. Text+Audio	Null	
	Schroeder et al., 2001	IPT-Desktop vs. IPT-IPT	IPT-Desktop < IPT-IPT	
	Sallnäs, 2005 (Study 1)	Text vs. Audio vs. Audio+Video	Others > Text	
	Sallnäs, 2005 (Study 2)	Audio vs. Audio+Video	Null	
	Sallnäs, 2005 (Study 2)	Web vs. CVE	Null	
	Slater et al., 1999	Desktop vs. HMD	Null	
	Slater et al., 2000	Desktop vs. HMD	Null	
	Steed et al., 1999	Desktop vs. HMD	Null	
	Wideström et al., 2000	Desktop vs. CAVE	Null	
	Yoo and Alavi, 2001	Audio vs. Audio+Video	+	
	Zhan and Mei, 2013	CMC vs. FtF	+	
Visual representation	Bailenson et al., 2001	Photographic realism	Null	
	Bailenson et al., 2001	Behavioral realism (Mutual gaze)	+	
	Bailenson et al., 2003 (Study 1)	Behavioral realism (Mutual gaze)	+	
	Bailenson et al., 2003 (Study 2)	Behavioral realism (Mutual gaze)	+	
	Bailenson et al., 2005	Match between visual and behavioral realism	+	
	Bente et al., 2007 (Study 1)	Behavioral realism (Mutual gaze)	+	
	Bente et al., 2007 (Study 2)	Behavioral realism (Mutual gaze)	Inverted U*	*Moderated by gender
	Bente et al., 2008	Photographic realism (Low vs. High fidelity avatar)	Null	
	Casanueva and Blake, 2001 (Study 2)	Behavioral realism (Static vs. Dynamic)	+	
	Choi et al., 2001	Absent vs. Present	+	
	Clayes and Anderson, 2007	Photographic realism (Avatar icon vs. Video image)	Null	
	Croes et al., 2016	Invisible vs. Visible	+	
	Dalzel-Job, 2014 (Study 2)	Behavioral realism (Mutual gaze)	Null	
	Fortin and Dholakia, 2005	Vividness	+	
	Garau et al., 2003	Match between visual and behavioral realism	+	
	Garau et al., 2005	Behavioral realism (static vs. moving vs. responsive vs. talking)	Static < responsive	No other significant differences
	Gong, 2008	Anthropomorphism (low vs. medium vs. high vs. real human)	+	
	Guadagno et al., 2007 (Study 1)	Behavioral realism	+	
	Guadagno et al., 2007 (Study 2)	Behavioral realism	+	
	Kang and Gratch, 2014	Behavioral realism (High, Low, None)	Null	
	Kang and Watt, 2013	Anthropomorphism (Low vs. High)	+	
	Kang and Watt, 2013	Behavioral realism (Static vs. Dynamic)	Null	
	Kang et al., 2008	Behavioral realism (Static vs. Dynamic)	+	
	Kang et al., 2008	Visual realism (Graphic vs. Video)	+	
	Kim and Sundar, 2012	Absent vs. Present (virtual character)	−*	*Moderated by interactivity
	Kim et al., 2004	Pointer (Absent vs. Present)	−	
	Kim et al., 2013b	Absent vs. Present	+	
	Lee et al., 2005	Behavioral realism (Low vs. High developmental capacity)	+	
	Meyer and Lohner, 2012	Absent vs. Present	+	
	Nowak and Biocca, 2003	Anthropomorphism (No image vs. Low vs. High)	No image/High < Low	
	Pan et al., 2008	Blushing behavior (non vs. cheeks vs. whole face)	Others < Whole Face	
	Park and Sundar, 2015	Absent vs. Picture vs. Emoticon	Absent/Picture < Emoticon	
	Qiu and Benbasat, 2005	3D Avatar (Absent vs. Present)	Null	
	Shahid et al., 2012	Behavioral realism (Mutual gaze)	+	
	Vishwanath, 2016 (Study 2)	Absent vs. Present	+	
	von der Pütten et al., 2010	Behavioral realism	+	
	Wu et al., 2014	Behavioral realism (Static vs. Dynamic)	+	
	Xu, 2014	Absent vs. Present (profile picture)	+	
Interactivity	Fortin and Dholakia, 2005	Low vs. Medium vs. High interactivity	+^*^	*Moderated by Need-for-Cognition
	Garau et al., 2005	Responsiveness	+	
	Han et al., 2016	Machine & person interactivity	+	
	Lee et al., 2007	Not interactive (offline quiz) vs. Interactive (online quiz)	+	
	Lee and Shin, 2012	Low vs. High interactivity	+^*^	*Moderated by affiliative tendency
	Lim and Lee-Won, 2017	Monologic vs. Dialogic	+	
	Nowak et al., 2009	Synchronocicity (Low vs. High)	+	
	Park and Sundar, 2015	Synchronocicity (Low vs. Medium vs. High)	Low/Medium < High	
	Phillips and Lee, 2005 (Study 3)	None vs. Simple vs. Complex	None/Simple < Complex	
	Qin et al., 2013	Synchronocicity (Haptic packet data loss: 0.3 vs. 0.2 vs. 0.1 vs. none)	+	
	Rauh and Renfro, 2004	Synchronocicity (No feedback delay vs. Feedback delay)	Null	
	Rauwers et al., 2016	Internal communication features: Absent vs. Present	+	Null for external communication features
	Shimoda, 2007	Generic vs. Tailored vs. Feedback-driven message	Null	
	Skalski and Tamborini, 2007	Not interactive vs. Interactive	+	
	Zelenkauskaite and Bucy, 2009	Passive vs. Interactive	+	
Haptic feedback	Basdogan et al., 2000	Absent vs. present	+	
	Chellali et al., 2011	Absent vs. present	+	
	Giannopoulos et al., 2008	Absent vs. present	+	
	Jordan et al., 2002	Absent vs. Present	+	
	Kim et al., 2004	Absent vs. present	+	
	Lee et al., 2017	Sound vs. Sound + Vibrotactile Feedback	+	No differences found between No Sound vs. Sound/Sound+Vibrotactile Feedback
	Lee et al., 2018	Absent vs. Present	+	
	Nam et al., 2008	Absent vs. Present	+	
	Sallnäs, 2010	Absent vs. Present	+	
	Sallnäs et al., 2000	Absent vs. present	Null	
Depth cues	Ahn et al., 2014	Stereoscopy (Mono vs. Stereo)	+	
	Kim et al., 2012 (Study 1)	Mono vs. Motion parallax vs. Stereo+Motion parallax	Mono < Motion parallax/Stereo+Motion parallax	
	Kim et al., 2012 (Study 2)	Mono vs. Motion parallax vs. Stereo+Motion parallax	Mono < Motion parallax/Stereo+Motion parallax	
	Mühlbach et al., 1995 (Study 1)	Stereoscopy (Mono vs. Stereo)	+	
	Takatalo et al., 2011	Mono vs. Medium stereo separation vs. High stereo separation	Inverted U	
Audio quality	Christie, 1974	Singlespeaker (speakerphone/high-fidelity speaker phone) vs. Multi-speaker	+	
	Dicke et al., 2010	Monophonic vs. Stereophonic vs. Binaural	Binaural > Mono/Stereo	
	Skalski and Whitbred, 2010	Two-Channel Sound vs. Surround Sound	+	
Display	Ahn et al., 2014	One 55-inch screen vs. Three 55-inch screens	+	
	Bracken, 2005	Image quality (NTSC vs. HDTV)	+	
	James et al., 2011	30-inch LCD screen vs. rear-projection system on 13-foot dome	Null	
	Skalski and Whitbred, 2010	Image quality (Standard vs. High Definition)	Null	
Other	Chuah et al., 2013 (Study 1)	Low vs. High physicality	+*	*Moderated by plausibility
	Hayes, 2015	Static display vs. Motion control by tracking (Kinect)	Null	Positive for only for a few items
	Heidicker et al., 2017	No tracking vs. Tracking vs. Tracking+ Inverse Kinematics	Null	Positive for only for two sub-factors
	Hills et al., 2005	One view point vs. Multiple view points	–	
	Lee et al., 2016	Incidental movement of real-virtual object (Absent vs. Present)	+	
	Lee et al., 2006a (Study 1)	Virtual social robot vs. Physical social robot	+	
	Lee et al., 2006a (Study 2)	Virtual social robot vs. Physical social robot	−	Tactile interaction restricted
	Li et al., 2016	Human vs. robot virtual lecturer	–	
	Oh et al., 2016	“Jaw flap” vs. Facial tracking vs. Exaggerated facial tracking	“Jaw flap”/Facial tracking < Exaggerated facial tracking	
	Tanaka et al., 2015 (Study 1)	Low vs. High Physicality	+	
	Wu et al., 2015	Virtual bowling (exergame) vs. Physical bowling	+	
	Zibrek et al., 2017(Study 2)	Self-move vs. Other-move	Null	
**CONTEXT**				
Personality/Traits of virtual human	Al-Natour et al., 2011	Match between virtual shopping assistant and participant strategy	+	
	Aymerich-Franch et al., 2012	Match between participant's and avatar's voice	+	
	Bailenson and Yee, 2005	Mimicry	+	
	Gong et al., 2007 (Study 1)	Group identity (Mismatch vs. Match)	+*	*Moderated by identification
	Gong et al., 2007 (Study 2)	Group identity (Mismatch vs. Match)	+*	*Moderated by identification
	Guadagno et al., 2011	Perceived empathy	+	
	Han et al., 2016	Self-disclosure	+	
	Jin, 2012 (Study 2)	Match between physical and virtual other	+	
	Kang and Gratch, 2014	Self-disclosure (None vs. Low vs. High)	Low/None < High	
	Kim and Timmerman, 2018	Supportive feedback (Not supportive vs. supportive)	+	
	Kothgassner et al., 2014	Social inclusion (exclusion vs. inclusion)	Null	
	Kothgassner et al., 2017	Social inclusion (exclusion vs. inclusion)	Null	
	Lee and Nass, 2005 (Study 1)	Match between computer agent and participant personality	+	
	Lee and Nass, 2005 (Study 2)	Match between content & personality manifested by voice	+	
	Lee and Oh, 2012 (Study 1)	Impersonal vs. personal disclosure	+	
	Lee et al., 2006b	Match between robot and participant personality	+	
	McGregor, 2018	Impersonal vs. personal disclosure	+^*^	* Moderated by group identity and target gender
	Qiu and Benbasat, 2010	Same ethnicity vs. Different ethnicity	+^*^	*Moderated by gender
	Qiu and Benbasat, 2010	Same gender vs. Different gender	Null	
	Verhagen et al., 2014	Expert	+^*^	*Moderated by task type
	Verhagen et al., 2014	Friendly	+^*^	*Moderated by task type
	Verhagen et al., 2014	Smiling	Null	
	Xu and Lombard, 2017	Group identification	+	
Agency	Appel et al., 2012	Agent vs. Avatar	+	
	Bailenson et al., 2003 (Study 2)	Agent vs. Avatar	+	
	Dalzel-Job, 2014 (Study 2)	Agent vs. Avatar	Null	
	Felnhofer et al., 2018	Agent vs. Avatar	Null	
	Gajadhar et al., 2008	Agent vs. Avatar	+	
	Guadagno et al., 2007 (Study 2)	Agent vs. Avatar	+^*^	*Moderated by virtual human gender
	Hoyt et al., 2003	Agent vs. Avatar	+	
	Kothgassner et al., 2014	Agent vs. Avatar	Null	
	Kothgassner et al., 2017	Agent vs. Avatar	Null	
	Nowak and Biocca, 2003	Agent vs. Avatar	Null	
	Peña et al., 2017	Agent vs. Avatar	+	
	von der Pütten et al., 2010	Agent vs. Avatar	Null	
Physical proximity	Croes et al., 2016	Same room vs. Different rooms	+	
	Gajadhar et al., 2008	Same room vs. Different rooms	+	
	Hatta and Ken-ichi, 2008	Remote vs. close	+^*^	*Moderated by visibility
	Järvelä et al., 2016	Same room vs. Different rooms	+	
	Jung et al., 2017	Distant vs. Close (geolocation proximity)	+	
Task type	de Greef, 2014	Complex vs. Simple	+^*^	*Moderated by relationship
	Herrewijn and Poels, 2015	Be observer vs. Be Player vs. Collaborate	Observe/Play < Collaborate	
	Kim et al., 2013a	Human as care-giver vs. Robot as care-giver	+	
	Wu et al., 2015	Competitive vs. Collaborative	Null	
Social cues	Choi and Kwak, 2017 (Study 2)	Number of remote senders (single vs. multiple)	+	
	Daher et al., 2016	Exposure to other person interacting with VH (No vs. Yes)	+	
	Kim, 2016	Number of different voices (single vs. multiple)	+	
	Kim and Sundar, 2014	Online buddy (Absent vs. Present)	-	
	Lee and Nass, 2004 (Study 1)	Single voice vs. Multiple voices	+	
	Lee and Nass, 2004 (Study 2)	Single voice vs. Multiple voices	+	
	Lee et al., 2005	Number of participants (individual vs. group)	Null	
	Robb et al., 2016	Presence of human teammate (No vs. Yes)	Null	Null main effect, but significant interaction with role of virtual human
	Robb et al., 2016	Role of virtual human (anesthesiologist vs. surgeon)	+^*^	*Only when there was no human teammate
Identity cues	Choi and Kwak, 2017 (Study 1)	Telepresence robot: Low identity cues vs. High identity cues	+/−	Higher for robot, lower for remote sender
	Choi and Kwak, 2017 (Study 2)	Telepresence robot: Low identity cues vs. High identity cues	+/−	Higher for robot, lower for remote sender
	Feng et al., 2016	No personal cues vs. Name+Picture	+	
	Li et al., 2015	Non-name ID vs. Picture+name ID	+	
	Schumann et al., 2017	Non-name ID vs. Picture+name ID	+	
Other	Alghamdi et al., 2016 (Study 1)	Multiple vs. Integrated communication channels	+	
	Alghamdi et al., 2016 (Study 2)	Multiple vs. Integrated communication channels	+	
	Bouchard et al., 2013	Relationship (Virtual animal vs. Unknown VH vs. Known VH)	+	
	Feng et al., 2016	Gender of VH	Null	
	Horvath and Lombard, 2010	No social pleasantries & picture vs. Social pleasantries & picture	+	
	Jin, 2011	Match between regulatory strategy and task	+	
	Kang and Watt, 2013	Non-anonymous vs. Anonymous partner	-	
	Kim et al., 2016	Implausible vs. Plausible VH behavior	+	
	Kim et al., 2017	Implausible vs. Plausible VH behavior	+	
	Yoo and Alavi, 2001	Group cohesion (groups without vs. with a history)	+	
**INDIVIDUAL**				
Demographic variables	Bailenson et al., 2006	Gender (Male vs. Female)	+	
	Bracken, 2005	Gender (Male vs. Female)	Null	
	Cho et al., 2015	Gender (Male vs. Female)	Null	
	de Greef, 2014	Gender (Male vs. Female)	Null	
	de Greef and Ijsselsteijn, 2001	Gender (Male vs. Female)	+	
	Felnhofer et al., 2014	Gender (Male vs. Female)	Null	
	Giannopoulos et al., 2008	Gender (Male vs. Female)	+	
	Guadagno et al., 2007 (Study 1)	Gender (Male vs. Female)	Null	
	Hauber et al., 2005	Gender (Male vs. Female)	Null	
	Johnson, 2011	Gender (Male vs. Female)	+	
	Lim and Richardson, 2016	Gender (Male vs. Female)	Null	
	Lowden and Hostetter, 2012	Gender (Male vs. Female)	+	
	Nowak, 2003	Gender (Male vs. Female)	+	
	Qin et al., 2013	Gender (Male vs. Female)	+	
	Qiu and Benbasat, 2010	Gender (Male vs. Female)	Null	
	Richardson and Swan, 2003	Gender (Male vs. Female)	+	
	Thayalan et al., 2012	Gender (Male vs. Female)	+	
	Cho et al., 2015	Age	Null	
	Felnhofer et al., 2014	Age	Null	
	Hauber et al., 2005	Age	Null	
	Kim et al., 2004	Age	–	
	Lim and Richardson, 2016	Age	Null	
	Richardson and Swan, 2003	Age	Null	
	Siriaraya and Ang, 2012	Age	–	
Psychological traits	Cortese and Seo, 2012	Communication Apprehension	–	
	Giannopoulos et al., 2008	Shyness	–	
	Jin, 2010	Interdependent self-construal	+	
	Kim et al., 2013a	Immersive Tendency	+	
	Kim et al., 2013a	Need to Belong	+	
	Kim et al., 2016	Extraversion	+	
	Lee et al., 2006a	Loneliness	+	
	Lee and Shin, 2014	Transportability	+^*^	*Moderated by modality
Other	Cho et al., 2015	Epistemological Belief (Simple vs. Complex)	+	
	Gimpel et al., 2016	Channel competence (experience comfort with medium)	+	
	Tanaka et al., 2015 (Study 2)	Previous interaction experience (No vs. Yes)	+^*^	*Moderated by physicality

**Table 2:** Summary of study information.

**Reference**	**Social presence measurement**	**Task(s)**	**Target (AP, CA)**	**Location**
Ahn et al., 2014	Temple Presence Inventory (Lombard et al., 2009)	View a virtual character on a screen	CA	Korea
Al-Natour et al., 2011	Gefen and Straub, 2003	Online shopping task	CA	Canada
Alge et al., 2003	Custom construct	Collaborate in teams of 3 on two tasks (brainstorming and solution-seeking)	AP	USA
Alghamdi et al., 2016 (Study 1 & 2)	Short et al., 1976	Tidy up a virtual house	AP	New Zealand
Appel et al., 2012	Bailenson et al., 2001; Networked Minds Questionnaire (Biocca et al., 2001)	Interact with agent (but framed as agent or avatar)	CA	USA
Axelsson et al., 2001	Custom construct	Complete a Rubik's cube-type puzzle	AP	Sweden
Aymerich-Franch et al., 2012	Nowak and Biocca, 2003	Give a speech to a virtual audience	CA	USA
Bailenson and Yee, 2005	Slater et al., 2000	Listen to agent presentation	CA	USA
Bailenson et al., 2001	Custom construct	Walk up to virtual human, read and memorize information on front/back tags	CA	USA
Bailenson et al., 2003 (Study 1 & 2)	Custom construct	Approach virtual human (Study 1)/Observe virtual human approach participants (Study 2)	CA	USA
Bailenson et al., 2005	Custom construct	Look at virtual agent	CA	USA
Bailenson et al., 2006	Networked Minds Questionnaire (Biocca et al., 2001)	Interact with partner & Emoting task	AP	USA
Basdogan et al., 2000	Custom construct	Move a ring with the help of a partner without touching the wire	AP	USA
Bente et al., 2007 (Study 1 & 2)	Networked Minds Questionnaire (Biocca et al., 2001)	Get-acquainted task	AP	Germany
Bente et al., 2008	Biocca et al., 2001; Nowak, 2001; Kumar and Benbasat, 2002; Tu, 2002	Hire the most suitable job candidate (Management decision task) (hire most suitable job candidate)	AP	Germany
Bouchard et al., 2013	Gerhard et al., 2001; Bailenson et al., 2005	Interact with a virtual cat, view virtual humans in pain	CA	Canada
Bracken, 2005	Lombard et al., 2000	Watch a video (The Beauty of Japan)	AP	USA
Casanueva and Blake, 2001 (Study 1 & 2)	Custom construct	In groups of 3 participants, read a story and collaboratively rank the characters	AP	South Africa
Chellali et al., 2011	Not reported	Perform a needle insertion task in dyads after training session	AP	France
Cho et al., 2015	Wei and Chen, 2012	Take online course on Second Life	AP	Singapore
Choi and Kwak, 2017 (Study 1 & 2)	Short et al., 1976; Nowak and Biocca, 2003	Engage in a video call with a remote participant using a telepresence robot	AP	Korea
Choi et al., 2001	Short et al., 1976; Lombard, 1995	Navigate an advertising website	CA	USA
Chuah et al., 2013	Bailenson et al., 2003	Anesthesiologists interact with two embodied conversational agents (nurse & patient's daughter)	CA	USA
Christie, 1974	Custom construct	Discuss a modern business issue	AP	UK
Clayes and Anderson, 2007	Short et al., 1976	Participates complete focused and non-focused tasks in groups of three people	AP	Scotland
Cortese and Seo, 2012	Networked Minds Questionnaire (Biocca et al., 2001)	View news website and discuss issues	AP	USA
Croes et al., 2016	Nowak and Biocca, 2003	Get-acquainted task	AP	The Netherlands
Daher et al., 2016	Harms and Biocca, 2004	Play a guessing game	CA	USA
Dalzel-Job, 2014 (Study 2)	Custom construct	Carry out 10 tasks in a virtual environment with a partner	AP	Scotland
de Greef, 2014	IPO-Social Presence Questionnaire (de Greef and Ijsselsteijn, 2001)	Select pictures with partner based on instructions	AP	The Netherlands
de Greef and Ijsselsteijn, 2001	IPO-Social Presence Questionnaire (de Greef and Ijsselsteijn, 2001)	Use a PhotoShare application with partner	AP	The Netherlands
DeSchryver et al., 2009	Richardson and Swan, 2003	Participate in online discussion forum for psychology class	AP	USA
Dicke et al., 2010	Custom construct	Listen to an audio recording of multiple speakers	AP	Finland
Felnhofer et al., 2014	Bailenson et al., 2003	Navigate in a café in an IVE and interact with a waiter and a stranger	CA	Austria
Felnhofer et al., 2018	Bailenson et al., 2003	Navigate in a café in an IVE and interact with a waiter and a stranger	CA	Austria
Feng et al., 2016	Lee and Nass, 2005	Read supporter seeker's profile and respond on an online forum	AP	USA
Fortin and Dholakia, 2005	Short et al., 1976	View online ad and surf website	CA	USA
Francescato et al., 2006	Cuddetta et al., 2003	Complete small-group exercises as part of a seminar series	AP	Italy
Gajadhar et al., 2008	Social Presence in Gaming Questionnaire (IJsselsteijn et al., 2008)	Play game with partner	AP	The Netherlands
Garau et al., 2003	Custom construct	Participate in a role-playing negotiation task	AP	UK
Garau et al., 2005	Custom construct	Enter virtual room and observe surroundings	CA	UK
Giannopoulos et al., 2008	Basdogan et al., 2000	Solve a jigsaw puzzle with a partner	AP	Spain
Gimpel et al., 2016	Nowak and Biocca, 2003	Interact with a digital service agent (while applying for fictitious credit card)	AP	Germany
Gong, 2008	Short et al., 1976	Interact with virtual agent on how to respond to dilemma scenarios	CA	USA
Gong et al., 2007 (Study 1 & 2)	Short et al., 1976	Interact with virtual agent on an e-commerce website	CA	USA
Guadagno et al., 2007 (Study 1 & 2)	Swinth and Blascovich, 2001	Listen to agent presentation	CA	USA
Guadagno et al., 2011	6-item questionnaire (details not reported)	Interact with a virtual peer counselor	CA	USA
Han et al., 2016	Gefen and Straub, 2003	View (fictitious) corporate Twitter accounts	AP	Korea
Hatta and Ken-ichi, 2008	Short et al., 1976	Negotiate on the price of a used car	AP	Japan
Hauber et al., 2005	Short et al., 1976; Nowak and Biocca, 2003	Desert survival task	AP	New Zealand
Hauber et al., 2006	Short et al., 1976	Collaborative photo-matching task	AP	New Zealand
Hauber et al., 2012	Short et al., 1976	Collaborative celebrity-quote matching task	AP	New Zealand
Hayes, 2015	Bailenson et al., 2006	Deliver a lesson to virtual students	CA	USA
Heidicker et al., 2017	Biocca et al., 2001	Desert survival task	AP	Germany
Heldal et al., 2005	Custom construct	Complete a Rubik's cube-type puzzle	AP	Sweden
Herrewijn and Poels, 2015	Social Presence in Gaming Questionnaire (IJsselsteijn et al., 2008)	Play a multiplayer game	AP	Belgium
Hills, 2005 (Study 1)	Networked Minds Questionnaire (Short et al., 1976; Biocca et al., 2001)	Desert survival task	AP	New Zealand
Hills, 2005 (Study 2)	Short et al., 1976	Build a virtual house with a partner	AP	New Zealand
Hills et al., 2005	Short et al., 1976	Evaluate five house designs	AP	New Zealand
Homer et al., 2008	Kim and Biocca, 1997	Viewed computer-based multimedia presentation of lecture	AP	USA
Horvath and Lombard, 2010	Temple Presence Inventory (Lombard et al., 2009)	Test an interactive website for the submission of college admission application	CA	USA
Hoyt et al., 2003	Custom construct	Categorization task & pattern recognition task	CA	USA
James et al., 2011	Custom construct	Mining operators collaborate to move a mining vehicle through a maze	AP	Australia
Järvelä et al., 2016	Networked Minds Questionnaire (Harms and Biocca, 2004)	View a video	AP	Finland
Jin, 2009	Custom construct	View a virtual Apple store representative agent	CA	USA
Jin, 2011	Lee et al., 2006a,b	Interact with health consultant avatar	AP	USA
Jin, 2010	Lee et al., 2006a,b	Interact with a recommendation agent on Second Life	CA	USA
Jin, 2012 Study 2	Lee et al., 2006a,b	FtF communication, followed by Avatar-to-Avatar communication	AP	USA
Johnsen and Lok, 2008	Bailenson et al., 2005	Interview virtual patient	CA	USA
Johnson, 2011	Custom construct	Take online course	AP	USA
Jordan et al., 2002	Basdogan et al., 2000	Lift a cube with a virtual partner and keep it off the “ground” for as long as possible	AP	UK/USA
Jung et al., 2017	Custom construct	View online dating site profile	AP	USA
Kang and Gratch, 2014	Short et al., 1976	Interview-style interaction	CA	USA
Kang and Watt, 2013	Custom construct	Interact with partner on a mobile phone	AP	USA
Kang et al., 2008	Nowak and Biocca, 2003	Interact with partner on a mobile phone	AP	USA
Kim, 2016	Lee et al., 2006a,b	Listen to information about local weather, traffic, and events	CA	China
Kim and Sundar, 2012	Lee et al., 2006a,b	Browse sunscreen company website	CA	USA
Kim and Sundar, 2014	Gefen and Straub, 2003	Participate in an interactive online health community	AP	USA
Kim and Timmerman, 2018	Short et al., 1976; Lombard et al., 2000	Play an exergame (Nintendo Wii Fit Hula Hoop game)	CA	USA
Kim et al., 2014	Short et al., 1976	Online chat (listen to a story)	AP	USA
Kim et al., 2013a	Lee et al., 2006a,b	Interact with a Nao robot	CA	Korea
Kim et al., 2013b	Networked Minds Questionnaire (Biocca et al., 2001)	Interact with a partner in an online apparel store and choose an item	AP	Korea
Kim et al., 2004	Basdogan et al., 2000	Lift a box with a virtual partner	AP	UK
Kim et al., 2012 (Study 1)	Nowak and Biocca, 2003	View a virtual human and indicate where he/she is looking or pointing	AP	Canada
Kim et al., 2012 (Study 2)	Nowak and Biocca, 2003	View a virtual instructor in a yoga pose and instruct a partner to reproduce the pose	AP	Canada
Kim et al., 2016	Bailenson et al., 2003; Harms and Biocca, 2004	Answer questions from a virtual human (MBTI personality test)	CA	USA
Kim et al., 2017	Bailenson et al., 2003	Answer questions from a virtual human (MBTI personality test)	CA	USA
Kothgassner et al., 2014	Bailenson et al., 2003	Play a ball-tossing game	CA	Austria
Kothgassner et al., 2017	Bailenson et al., 2003	Play a ball-tossing game	CA	Austria
Lee, 2013	Nowak and Biocca, 2003; Lee and Nass, 2005	View politician on Twitter or on television	AP	Korea
Lee and Jang, 2013 (Study 1 & 2)	Lee and Nass, 2005	View politician's Twitter page or newspaper interview	AP	Korea
Lee and Nass, 2004	Custom construct	Listen to online reviews	CA	USA
Lee and Nass, 2005 (Study 1 & 2)	Custom construct	Listen to online reviews	CA	USA
Lee and Oh, 2012 (Study 1)	Nowak and Biocca, 2003	View politician's Twitter page	AP	Korea
Lee and Shin, 2012	Nowak and Biocca, 2003; Lee and Nass, 2005	View politician's Twitter page	AP	Korea
Lee and Shin, 2014	Nowak and Biocca, 2003; Lee and Nass, 2005	View politician's Twitter page or newspaper interview	AP	Korea
Lee et al., 2005	Biocca et al., 2001	Interact with an Aibo robot	CA	USA
Lee et al., 2006a	Custom construct	Interact with an Aibo robot	CA	USA
Lee et al., 2006b (Study 1 & 2)	Custom construct	Interact with virtual or physical social robot without (Study 1) or without (Study 2) tactile restrictions	CA	USA
Lee et al., 2007	Custom construct	Participate in an educational quiz game	AP	Korea
Lee et al., 2016	Harms and Biocca, 2004	Play 20 questions with a virtual human	CA	USA
Lee et al., 2017	Bailenson et al., 2003	Observe a virtual human walk, approach the participant, and leave	CA	USA
Lee et al., 2018	Basdogan et al., 2000; Bailenson et al., 2003	Walk around a virtual or real human that is engaging in various behaviors (standing, jumping, walking)	CA	USA
Li et al., 2015	Lee and Nass, 2005	Read a support-seeking post and type/post responses	AP	USA
Li et al., 2016	Lee et al., 2006a,b	Watch an online lecture	CA	USA
Lim and Lee-Won, 2017	Nowak and Biocca, 2003; Lee and Nass, 2005; Lee and Shin, 2014	View a (fictitious) food company's Twitter feed	AP	USA
Lowden and Hostetter, 2012	Hostetter and Busch, 2006	Answer survey regarding videoconferencing experience	AP	USA
McGregor, 2018	Lee and Oh, 2012	View a screenshot of a political candidate's Twitter feed	AP	USA
Meyer and Lohner, 2012	Gunawardena, 1995; Swan, 2002	Watch an online news video	AP	USA
Moreno and Mayer, 2004	Custom construct	Receive a lesson on botany from computerized agent	CA	USA
Mühlbach et al., 1995 (Study 1)	Custom construct	Collaborative decision-making task and negotiating task via videoconference	AP	Germany
Nam et al., 2008	Schroeder et al., 2001	Play air hockey game with remote partner	AP	USA
Nowak, 2003	Short et al., 1976	Desert survival task with text-based CMC	AP	USA
Nowak and Biocca, 2003	Custom construct	Get to know partner and compete in a virtual scavenger hunt	CA	USA
Nowak et al., 2009	Nowak and Biocca, 2003	Prepare an oral report in groups of 3-4 students over 5 weeks	AP	USA
Oh et al., 2016	Networked Minds (Harms and Biocca, 2004)	Play 20 questions and get acquainted with a virtual partner	AP	USA
Pan et al., 2008	Custom construct	Listen to agent presentation	CA	UK
Park and Sundar, 2015	Networked Minds (Harms and Biocca, 2004)	Interact with a customer service agent	CA	Korea
Peña et al., 2017	Networked Minds (Harms and Biocca, 2004)	Play a video game in a single-player or multi-player mode	CA & AP	Korea
Phillips and Lee, 2005 (Study 3)	Choi et al., 2001	View website with spokes-character	CA	USA
Qin et al., 2013 (Study 2)	Witmer and Singer, 1998; Kim et al., 2004	Collaborate with a partner to complete a ring-moving task	AP	China
Qiu and Benbasat, 2005	Short et al., 1976	Browse online electronics store, interact with customer service agent, and purchase items	AP	Canada
Qiu and Benbasat, 2010	Gefen and Straub, 2003	Interact with product recommendation agent	CA	Canada
Rauh and Renfro, 2004	Short et al., 1976	Participants talk with each other about a set of topics using a videoconferencing system	AP	USA
Rauwers et al., 2016	Gefen and Straub, 2003; Lee et al., 2011	Interact with a digital magazine	CA	USA
Robb et al., 2016	Bailenson et al., 2003	Medical practitioners work with a virtual surgeon and a virtual anesthesiologist to prepare for surgery	CA	USA
Richardson and Swan, 2003	Custom construct	Take online course	AP	The Netherlands
Sallnäs, 2005 (Study 1)	Short et al., 1976 & Custom construct	Decision-making task	AP	Sweden
Sallnäs, 2010	(Short et al., 1976) modified	Pass cubes without audio communication	AP	Sweden
Sallnäs et al., 2000	(Short et al., 1976) modified	Perform multiple collaborative tasks with virtual blocks	AP	Sweden
Schroeder et al., 2001	Custom construct	Complete a Rubik's cube-type puzzle	AP	Sweden
Schumann et al., 2017 (Study 2)	Rüggenberg, 2007; Park and Sundar, 2015	Collaborate with a student from a different university (confederate) to develop ideas for an event	AP	Belgium
Shahid et al., 2012	Garau et al., 2001; Biocca and Harms, 2002	Play game with partner	AP	The Netherlands
Shimoda, 2007	Short et al., 1976	Participate in a multi-session online system that delivers messages that encourages smokers to quit	CA	USA
Siriaraya and Ang, 2012	Slater et al., 2000; Nowak and Biocca, 2003	Select avatar and interact with partner	AP	UK
Skalski and Tamborini, 2007	Nowak and Biocca, 2003	Listen to health information	CA	USA
Skalski and Whitbred, 2010	Lombard et al., 2009	Play a shooter game	CA	USA
Slater et al., 1999	Custom construct	Give a presentation to a virtual audience	CA	UK
Slater et al., 2000	Custom construct	Word puzzle & monitor group member (for some participants)	AP	UK
Steed et al., 1999	Custom construct	Collaborate in groups of three to carry out a puzzle-solving task.	AP	UK & Greece
Takatalo et al., 2011	Takatalo, 2002	Play a first-person driving game for 40 minutes	CA	Finland
Tanaka et al., 2015	Nakanishi et al., 2008	Talk with a remote partner about an issue	AP	Japan
Thayalan et al., 2012	Custom construct	Take online course	AP	Malaysia
Verhagen et al., 2014	Yoo and Alavi, 2001	Interact with virtual customer service agent	CA	The Netherlands
Vishwanath, 2016 (Study 2)	Slater et al., 1998	Simulated phishing attack	AP	Singapore
von der Pütten et al., 2010	(Bailenson et al., 2001) and Networked Minds Questionnaire (Biocca et al., 2001)	Interact with Rapport Agent	CA	USA
Wideström et al., 2000	Custom construct	Complete a Rubik's cube-type puzzle	AP	Sweden
Wu et al., 2014	Bailenson et al., 2003	Complete 4 nurse shifts for a virtual patient	CA	USA
Wu et al., 2015	Social Presence in Gaming Questionnaire (IJsselsteijn et al., 2008)	Bowl with a team on an exergame platform or with an indoor bowling set	AP	Singapore
Xu, 2014	Short et al., 1976	Read online reviews	AP	USA
Yoo and Alavi, 2001	Short et al., 1976	”Van Management“ task (Mennecke and Wheeler, 1993)	AP	USA
Zelenkauskaite and Bucy, 2009	Custom construct	Watch four videos of a politician	AP	USA
Zhan and Mei, 2013	Social Presence Inventory (Biocca and Harms, 2002)	Take a course online or offline	AP	China
Zibrek et al., 2017 (Study 1 & 2)	Bailenson et al., 2003	Approach a virtual character	CA	Ireland

*AP, Actual person; includes “fictitious” people if all of the virtual content was directly generated by an actual person (e.g., Twitter account of a fictitious politician)*.

*CA, Computer algorithm; includes instances wherein pre-programmed messages and/or animations were selected by a human controller (“Wizard of Oz” technique; Kim et al., 2016)*.

**Table 3:** Summary of publication impact factor^a^, sample size, and number of citations^b^ of reviewed studies.

**References**	**Publication outlet**	***N***	**Most recent impact factor**	**No. of Citations**
Ahn et al., 2014	Cyberpsychology, Behavior, and Social Networking	144	2.689	4
Al-Natour et al., 2011	Journal of the Association for Information Systems	181	2.839	61
Alge et al., 2003	Organizational Behavior and Human Decision Processes	198	2.259	322
Alghamdi et al., 2016 (Study 1 & 2)	Pacific Asia Conference on Information Systems	67 & 50	N/A	2
Appel et al., 2012	Advances in Human-Computer Interaction	90	N/A	25
Axelsson et al., 2001	Cyberpsychology and Behavior	44	2.689	39
Aymerich-Franch et al., 2012	International Workshop on Presence	51	N/A	12
Bailenson and Yee, 2005	Psychological Science	69	6.128	493
Bailenson et al., 2001	Presence: Teleoperators and Virtual Environments	50	0.426	357
Bailenson et al., 2003 (Study 1 & 2)	Personality and Social Psychology Bulletin	80 & 80	2.498	491
Bailenson et al., 2005	Presence: Teleoperators and Virtual Environments	146	0.426	237
Bailenson et al., 2006	Presence: Teleoperators and Virtual Environments	30	0.426	252
Basdogan et al., 2000	ACM Transactions on Computer-Human Interaction	10	0.972	432
Bente et al., 2007 (Study 1 & 2)	International Workshop on Presence	76 & 82	N/A	48
Bente et al., 2008	Human Communication Research	150	2.364	354
Bouchard et al., 2013	Cyberpsychology, Behavior, and Social Networking	42	2.689	30
Bracken, 2005	Media Psychology	95	2.574	168
Casanueva and Blake, 2001 (Study 1 & 2)	Annual Conference of the South African Institute of Computer Scientists and Information Technologists	18 & 18	N/A	49
Chellali et al., 2011	Interacting with Computers	60	0.809	24
Cho et al., 2015	Internet and Higher Education	128	5.847	26
Choi and Kwak, 2017 (Study 1 & 2)	Cognitive Systems Research	60 & 72	N/A	1
Choi et al., 2001	Journal of Interactive Advertising	210	N/A	139
Christie, 1974	European Journal of Social Psychology	36	N/A	N/A
Chuah et al., 2013	Presence: Teleoperators and Virtual Environments	23	0.426	18
Clayes and Anderson, 2007	International Journal of Human-Computer Studies	72	2.3	23
Cortese and Seo, 2012	Communication Research Reports	152	N/A	13
Croes et al., 2016	Computers in Human Behavior	210	3.536	6
Daher et al., 2016	IEEE Virtual Reality Conference	24	N/A	–
Dalzel-Job, 2014 (Study 2)	The University of Edinburgh	48	N/A	–
de Greef, 2014	International Workshop on Presence	42	N/A	–
de Greef and Ijsselsteijn, 2001	Cyberpsychology and Behavior	34	2.689	121
DeSchryver et al., 2009	Society for Information Technology and Teacher Education International Conference	31	N/A	140
Dicke et al., 2010	British Computer Society Interaction Specialist Group Conference	82	N/A	2
Felnhofer et al., 2014	Computers in Human Behavior	124	3.536	33
Felnhofer et al., 2018	Computers in Human Behavior	95	3.536	–
Feng et al., 2016	Communication Research	202	3.391	18
Fortin and Dholakia, 2005	Journal of Business Research	360	2.509	516
Francescato et al., 2006	Computers in Human Behavior	50	3.536	190
Gajadhar et al., 2008	International Conference of Fun and Games	2006-	0.402	125
Garau et al., 2003	Conference on Human Factors in Computing Systems (CHI)	48	N/A	311
Garau et al., 2005	Presence: Teleoperators and Virtual Environments	41	0.426	145
Giannopoulos et al., 2008	International Conference on Human Haptic Sensing and Touch Enabled Computer Applications	40	0.402	11
Gimpel et al., 2016	European Conference on Information Systems	528	N/A	5
Gong, 2008	Computers in Human Behavior	168	3.536	116
Gong et al., 2007 (Study 1 & 2)	Annual Conference of the International Communication Association	53 & 64	N/A	3
Guadagno et al., 2007 (Study 1 & 2)	Media Psychology	65 & 174	2.574	216
Guadagno et al., 2011	Computers in Human Behavior	38	3.536	45
Han et al., 2016	International Journal of Information Management	809	4.516	14
Hatta and Ken-ichi, 2008	Computers in Human Behavior	43	3.536	11
Hauber et al., 2005	International Workshop on Presence	42	N/A	76
Hauber et al., 2006	ACM Conference on Computer Supported Cooperative Work (CSCW)	30	N/A	57
Hauber et al., 2012	The Open Software Engineering Journal	36	N/A	7
Hayes, 2015	University of Central Florida	20	N/A	1
Heidicker et al., 2017	IEEE Symposium on 3D User Interfaces	18	N/A	2
Heldal et al., 2005	IEEE Virtual Reality Conference	220	N/A	31
Herrewijn and Poels, 2015	Computers in Human Behavior	121	3.536	12
Hills, 2005 (Study 1 & Study 2)	University of Otago	42 & 35	N/A	10
Hills et al., 2005	International Conference on Augmented Tele-Existence	35	N/A	9
Homer et al., 2008	Computers in Human Behavior	26 & 25	3.536	148
Horvath and Lombard, 2010	PsychNology Journal	189	N/A	27
Hoyt et al., 2003	Presence: Teleoperators and Virtual Environments	48	0.426	125
James et al., 2011	The Ergonomics Open Journal	10	N/A	1
Järvelä et al., 2016	Frontiers in Psychology	61	2.089	3
Jin, 2009	Cyberpsychology and Behavior	48	2.689	68
Jin, 2010	Presence: Teleoperators and Virtual Environments	179	0.426	23
Jin, 2011	Journal of Broadcasting and Electronic Media	101	1.773	75
Jin, 2012 (Study 2)	Computers in Human Behavior	148	3.536	27
Johnsen and Lok, 2008	IEEE Virtual Reality Conference	27	N/A	22
Johnson, 2011	Journal of Organizational and End User Computing	555	0.744	45
Jordan et al., 2002	International Workshop on Presence	20	N/A	32
Jung et al., 2017	Cyberpsychology, Behavior, and Social Networking	590	2.689	1
Kang and Gratch, 2014	Computers in Human Behavior	171	3.536	11
Kang and Watt, 2013	Computers in Human Behavior	196	3.536	15
Kang et al., 2008	Hawaii International Conference on System Sciences	126	N/A	31
Kim, 2016	Journal of Computer-Mediated Communication	100	4	6
Kim et al., 2014	Computers in Human Behavior	80	3.536	13
Kim et al., 2013a	Computers in Human Behavior	60	3.536	45
Kim et al., 2013b	Information and Management	80	3.89	68
Kim and Sundar, 2012	Computers in Human Behavior	93	3.536	60
Kim and Sundar, 2014	Computers in Human Behavior	100	3.536	24
Kim and Timmerman, 2018	Journal of Media Psychology	47	1.118	4
Kim et al., 2004	Presence: Teleoperators and Virtual Environments	20	0.426	146
Kim et al., 2012 (Study 1 & 2)	Conference on Human Factors in Computing Systems (CHI)	14 & 11	NA	79
Kim et al., 2016	International Conference on Artificial Reality and Tele-Existence	31	N/A	2
Kim et al., 2017	Computer Animation and Virtual Worlds	22	0.697	2
Kothgassner et al., 2014	International Workshop on Presence	48	N/A	–
Kothgassner et al., 2017	Computers in Human Behavior	45	3.536	4
Lee, 2013	Journal of Communication	183	3.729	34
Lee and Jang, 2013 (Study 1 & 2)	Communication Research	143 & 100	3.391	57
Lee and Nass, 2004	Human Communication Research	40	2.364	107
Lee and Nass, 2005 (Study 1 & 2)	Media Psychology	72 & 80	2.574	122
Lee and Oh, 2012(Study 1)	Journal of Communication	164	3.729	72
Lee and Shin, 2012	Cyberpsychology, Behavior, and Social Networking	264	2.689	84
Lee and Shin, 2014	Communication Research	217	3.391	54
Lee et al., 2005	Human Communication Research	40	2.364	78
Lee et al., 2006a	Journal of Communication	48	3.729	238
Lee et al., 2006b (Study 1 & 2)	International Journal of Human-Computer Studies	32 & 32	2.3	176
Lee et al., 2007	International Workshop on Presence	41	N/A	4
Lee et al., 2016	IEEE Virtual Reality Conference	20	N/A	11
Lee et al., 2017	IEEE Virtual Reality Conference	41	N/A	6
Lee et al., 2018	IEEE Transactions on Visualization and Computer Graphics	26	3.078	–
Li et al., 2015	Communication Quarterly	198	N/A	2
Li et al., 2016	Computers in Human Behavior	40	3.536	27
Lim and Lee-Won, 2017	Telematics and Informatics	128	3.789	5
Lowden and Hostetter, 2012	Computers in Human Behavior	157	3.536	16
McGregor, 2018	New Media & Society	1181	3.121	7
Meyer and Lohner, 2012	Annual Conference of the International Communication Association	120	N/A	–
Moreno and Mayer, 2004	Journal of Educational Psychology	48	4.433	317
Mühlbach et al., 1995 (Study 1)	Human Factors: The Journal of the Human Factors and Ergonomics Society	32	2.371	123
Nam et al., 2008	Computers in Human Behavior	36	3.536	27
Nowak, 2003	Media Psychology	42	2.574	57
Nowak and Biocca, 2003	Presence: Teleoperators and Virtual Environments	134	0.426	576
Nowak et al., 2009	Computers in Human Behavior	142	3.536	36
Oh et al., 2016	PLOS One	158	2.766	6
Pan et al., 2008	International Workshop on Presence	33	N/A	8
Park and Sundar, 2015	Computers in Human Behavior	108	3.536	17
Peña et al., 2017	Journal of Media Psychology	216	1.118	2
Phillips and Lee, 2005 (Study 3)	Journal of Current Issues and Research in Advertising	71	N/A	–
Qin et al., 2013 (Study 2)	Presence: Teleoperators and Virtual Environments	20	0.426	6
Qiu and Benbasat, 2005	International Journal of Human-Computer Interaction	72	1.259	159
Qiu and Benbasat, 2010	International Journal of Human-Computer Studies	188	2.3	83
Rauh and Renfro, 2004	Annual Conference of the International Communication Association	34	N/A	7
Rauwers et al., 2016	Computers in Human Behavior	195	3.536	3
Richardson and Swan, 2003	Journal of Asynchronous Learning Networks	97	N/A	1855
Robb et al., 2016	Frontiers in ICT	92	NA	1
Sallnäs, 2010	Haptics: Generating and Perceiving Tangible Sensations	18	0.402	17
Sallnäs, 2005 (Study 1)	Presence: Teleoperators and Virtual Environments	60	0.426	119
Sallnäs et al., 2000	ACM Transactions on Computer-Human Interaction	14	0.972	382
Schroeder et al., 2001	Computers and Graphics	132	1.2	150
Schumann et al., 2017 (Study 2)	Computers in Human Behavior	37	3.536	1
Shahid et al., 2012	Interacting with Computers	88	0.809	25
Shimoda, 2007	Annual Conference of the International Communication Association	51	N/A	–
Siriaraya and Ang, 2012	Interacting with Computers	60	0.809	29
Skalski and Tamborini, 2007	Media Psychology	235	2.574	88
Skalski and Whitbred, 2010	PsychNology Journal	74	N/A	66
Slater et al., 1999	IEEE Computer Graphics and Applications	10	1.64	192
Slater et al., 2000	Presence: Teleoperators and Virtual Environments	30	0.426	359
Steed et al., 1999	IEEE Virtual Reality Conference	60	N/A	88
Takatalo et al., 2011	Media Psychology	91	2.574	56
Tanaka et al., 2015	Frontiers in ICT	36 & 16	NA	11
Thayalan et al., 2012	Procedia-Social and Behavioral Sciences	51	N/A	7
Verhagen et al., 2014	Journal of Computer-Mediated Communication	296	4	39
Vishwanath, 2016 (Study 2)	Computers in Human Behavior	104	3.536	8
von der Pütten et al., 2010	Computers in Human Behavior	83	3.536	165
Wideström et al., 2000	International Conference on Collaborative Virtual Environments	88	N/A	32
Wu et al., 2014	IEEE Transactions on Visualization and Computer Graphics	22	3.078	17
Wu et al., 2015	Cyberpsychology, Behavior, and Social Networking	113	2.689	14
Xu, 2014	Computers in Human Behavior	152	3.536	72
Yoo and Alavi, 2001	Management Information Systems Quarterly	135	5.43	724
Zelenkauskaite and Bucy, 2009	International Workshop on Presence	67	N/A	2
Zhan and Mei, 2013	Computers and Education	257	4.538	86
Zibrek et al., 2017 (Study 1 & 2)	ACM Symposium on Applied Perception	38 & 18	N/A	2

a *Impact factor was retrieved from Web of Science Journal Citation Reports on August 19, 2018*.

b *Number of citations was retrieved from Google Scholar on August 19, 2018*.

## Materials and method

To collect studies that focused on the antecedents of social presence, we directly reviewed the archives of academic journals with a focus on virtual environments including *Computers in Human Behavior*; *Cyberpsychology, Behavior, and Social Networking*; *Journal of Computer-Mediated Communication*; *Presence: Teleoperators & Virtual Environments*; *Frontiers in Robotics and AI*, and conference proceedings from the *International Society for Presence Researchers (ISPR) Conference* and the *IEEE Conference on Virtual Reality*. We chose these outlets by selecting and expanding upon the outlets chosen in a recent meta-analysis on presence conducted by Cummings and Bailenson (2016). Based on concepts and terms that co-occurred frequently according to the subjective judgment of the researcher, we also conducted keyword searches in the *EBSCO Host Communication & Mass Media databases, PsycNET*, the *Temple University ISPR Telepresence Literature Refshare database*, and *Google Scholar*. Search terms included a combination of terms related to social presence, such as “social presence,” “co-presence,” “social richness,” “computers as social actors,” “virtual reality,” “virtual environments,” and “immersion” in addition to predictors that we identified during our search including “modality,” “HMD,” “realism,” “stereoscopy,” “haptics,” “audio,” “display,” “tracking,” “gender,” “agency,” and “proximity.”

Once the candidate studies were identified, we selected studies that (1) used at least one self-report measure of social presence (or the synonymous concept of co-presence); if social presence and co-presence were measured separately, we considered both measures in our review, (2) included experimental manipulations and/or questionnaire items (e.g., personality, gender, etc.) that were used to assess the predictors of social presence, and (3) conducted quantitative analyses to determine whether a predictor significantly influenced perceptions of social presence.

Studies that measured social presence with related but distinct constructs (e.g., interactivity, positive affect, social influence, telepresence, interpersonal attraction, electronic propinquity) were not included, as they do not uniquely measure the extent to which one feels as if she is present with a sentient being. Similarly, concepts that share theoretical similarities with, but do not uniquely measure social presence, were excluded. One significant concept that was not included due to this criterion was plausibility illusion (Slater, 2009). Plausibility illusion refers to the credibility of the events that are unfolding in the virtual environment. According to Slater (2009), plausibility illusion is orthogonal to the “sense of being there,” which is conceptualized as place illusion. Plausibility illusion shares similarities with social presence as it captures the extent to which the user feels that he or she is interacting with an actual social being (Biocca et al., 2003; Lee et al., 2006a). However, plausibility illusion also includes dimensions other than social presence because the concept simultaneously captures the credibility of various aspects of a virtual scenario, not just the virtual human (Slater et al., 2010).

**Figure 1:**
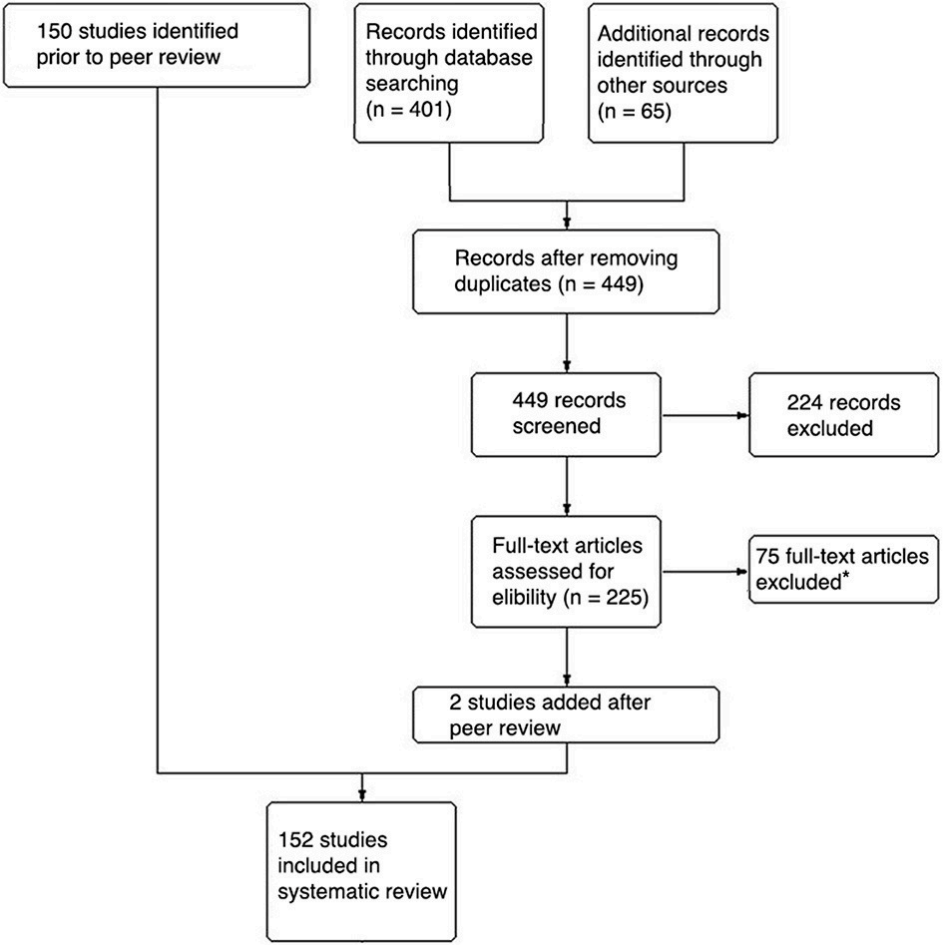
Flow chart of study identification. *Social presence is not a dependent variable: 27; No quantitative self-report measure of social presence: 24; Review article: 9; General presence is measured: 6; Work-in-progress: 3; Measure not reported: 1; Conference presentation of published article: 5.

Finally, while we are aware of the strengths of behavioral and physiological measures and limitations of self-report measures (Slater, 2004; Friedman et al., 2006), we did not include studies that exclusively used behavioral and/or physiological measures of social presence to reduce variance and maximize internal validity when comparing study findings. The criteria used to select studies were adapted from Cummings and Bailenson (2016) to fit the current context. Based on this process (Figure 1), we were able to identify 152 studies with 233 separate findings regarding the factors that can predict social presence. When discussing the results, we assumed that the findings of the studies were true and correct.

## Findings: what predicts social presence?

Considering that social presence was initially considered to be an inherent quality of a communication medium (Short et al., 1976), it is natural that a significant body of research explored how modality influences social presence. For similar reasons, the technological affordances that enable the reproduction of various social cues (e.g., presence of a visual representation, haptic feedback, etc.) have received considerable attention as potential antecedents of social presence. However, while earlier studies on the predictors of social presence focused almost entirely on immersive qualities, more recent studies also consider the impact of contextual and individual factors, perhaps as an acknowledgment of social presence as a subjective experience. The following sections will thus categorize and discuss the predictors of social presence using three overarching categories that emerged while conducting the systematic review: immersive qualities, contextual properties, and individual traits.

### Immersive qualities and social presence

#### General modality

Much of the earlier social presence research focused on how modalities with varying levels of immersion afford different levels of presence. It is important to note that while research on general modality does offer insight into how certain technological features (e.g., depth cues, display, stereoscopy) might influence social presence, it compares media that vary across multiple features, which makes it difficult to isolate the affordance(s) that influenced perceptions of social presence. This camp of research is well-aligned with the traditions of social presence theory (Short et al., 1976) and media richness theory (Daft and Lengel, 1986) in that they are grounded on the assumption that the technological qualities of a medium afford different levels of social presence. In their meta-analysis on the impact of immersion on telepresence, Cummings and Bailenson (2016) similarly found that general modality (e.g., comparing an HMD with head-tracking to a desktop computer) was one of the most frequently studied predictors of telepresence.

As can be seen in Table 1, research on the impact of modality on social presence to date most often compares (1) CMC with FtF communication, (2) text-based CMC with other forms of audiovisual modalities, and (3) immersive virtual environments with non-immersive virtual environments. Although it is less common, a small number of studies also compare different types of virtual environments (e.g., Heldal et al., 2005; Johnsen and Lok, 2008).

Because FtF interaction is considered to be the gold-standard for social presence (Biocca et al., 2001), a considerable amount of research compares FtF communication with CMC to determine how successful a given system is at establishing a social presence. Most of these studies found that communicators experience lower levels of social presence during CMC compared to FtF conversations. For example, Cortese and Seo (2012) found that CMC participants felt less social presence than FtF participants while they were discussing issues mentioned in a news article for 20 min. More specifically, the researchers operationalized social presence to assess both how sociable their partner was and how “co-located” they felt with their partner, and found that FtF communicators experienced higher levels of social presence compared to their CMC counterparts. Similar results were found in online learning contexts (Zhan and Mei, 2013) and decision-making scenarios (Biocca et al., 2001; Alge et al., 2003). One exception to this trend was Francescato et al. (2006) study, which found no differences in perceived social presence between students who completed a seminar series online, compared to those who completed the same seminar face-to-face. It is important to note, however, that participants completed the seminar series over a period of 2 months. This extended experiment period may be why the authors did not find a difference between CMC and FtF conditions. Just as Walther (1992) found that granting additional time to CMC interactants led to equally desirable communication outcomes as their FtF counterparts, the 2-month period employed by Francescato et al. (2006) may have been sufficient for both groups of participants to adapt their communication strategies to the given platforms and attain similar levels of social presence.

Studies that compared text-based CMC with more vivid forms of communication modalities (e.g., audio, video, avatar) also found that participants felt the lowest level of social presence when communicating via text-based CMC compared to “richer” forms of media, when given the same amount of time (e.g., Bente et al., 2008; Appel et al., 2012; Kim et al., 2013b). For example, Bente et al. (2008) measured how much social presence participants felt while selecting the best job candidate out of a pool of six applicants in a text chat, audio, audio with video, or avatar communication platform. They found that participants in the text chat condition felt significantly less social presence when compared with participants who communicated via other modalities. Similarly, studies that compared text-based CMC with modalities that offered audiovisual cues, such as videoconferencing (Sallnäs, 2005; Kim et al., 2014), avatar-mediated communication, and audio communication (Kim et al., 2013b) generally found that text-based CMC elicits lower social presence than modalities that offer additional audiovisual cues.

While audio and video modalities appear to have a clear advantage over text-based CMC, the strength of audiovisual modalities over audio-only modalities is less clear. Of the nine studies identified in Table 1 (de Greef and Ijsselsteijn, 2001; Yoo and Alavi, 2001; Sallnäs, 2005, Study 1 & 2; Bailenson et al., 2006; Bente et al., 2008; Homer et al., 2008; de Greef, 2014; Gimpel et al., 2016) that offered a comparison between audio-only and audio-video modalities, only four found that the addition of video increased perceptions of social presence (de Greef and Ijsselsteijn, 2001; Yoo and Alavi, 2001; de Greef, 2014; Gimpel et al., 2016). While the sample size is small (*n* = 9), these results suggest that linear increments of immersion do not necessarily lead to corresponding increases in social presence. Considering that two of the studies (de Greef and Ijsselsteijn, 2001; de Greef, 2014) that did find that adding video increased social presence required participants to complete a visual task, while the studies that did not find differences between the audio-only and audio-video conditions provided participants with tasks that had a weaker visual component (e.g., decision-making task, interview task), it is possible that the nature of the task moderates the benefits of adding video to audio. Table 2 shows details of these studies.

A small number of studies (e.g., Steed et al., 1999; Slater et al., 2000; Moreno and Mayer, 2004) have also compared immersive virtual platforms (e.g., HMD, cave automatic virtual environment; CAVE) with non-immersive ones (e.g., Desktop). While the literature shows a general consensus that immersive virtual environments are more likely to generate greater feelings of telepresence compared to non-immersive virtual platforms (Cummings and Bailenson, 2016), this does not appear to be the case for social presence. Among the 10 studies that we identified, only two studies found significant differences in social presence between an immersive platform and a non-immersive one (Schroeder et al., 2001; Heldal et al., 2005). These results, coupled with the fact that the addition of video does not consistently increase one's sense of social presence, suggest that once a threshold is met, increasing the immersive quality of a modality does not automatically lead to increased social presence. As such, it may be both theoretically and practically important to isolate features and explore the extent to which each feature does (or does not) contribute to increasing social presence to further understand the dimensions of immersion that affect social presence.

#### Visual representation

One of the unique features that influence social presence in virtual environments is the visual representation of the communication partner. Studies that focus on visual representations explore how the appearance of the partner in virtual reality influences one's sense of social presence. These studies generally manipulate (1) the presence or absence of a visual representation and (2) the visual realism of the virtual representation. Visual realism consists of photographic, anthropomorphic, and behavioral (or communicative) realism (Harris et al., 2009). Photographic and anthropomorphic realism both pertain to the appearance of the virtual representation; the former assesses how “realistic” it appears, while the latter refers to how “humanlike” it is. In contrast, behavioral realism is defined as the extent to which the virtual representation behaves in the way an actual person would behave (e.g., blink naturally, shift positions, “breathe,” etc.).

While there are a few exceptions (e.g., Qiu and Benbasat, 2005; Kim and Sundar, 2012), most of the current evidence indicates that people feel higher levels of social presence when there is a visual representation available, as can be noted in Table 1. For example, participants who were able to see their partner's avatar reported higher levels of social presence compared to those who spoke with an “invisible” partner after they shopped for clothes together in a virtual shopping mall (Kim et al., 2013b). Another study (Feng et al., 2016) similarly found that participants felt greater social presence toward online support-seekers who provided a profile picture compared to those who did not. Furthermore, participants were more likely to give responses that reflected an awareness of and adaptation to the support-seeker and his/her context (person-centeredness) when there was a profile picture available, an effect that was partially mediated by social presence.

In addition to the impact of providing a visual representation, studies have also examined how the extent to which a visual representation behaves like an actual person (i.e., behavioral realism) affects social presence. Behavioral realism can be operationalized by the complete absence or presence of nonverbal behavior (animations) or how much the virtual human's nonverbal behavior is consistent with actual humans (e.g., presence or absence of eye gaze). Studies generally show that behavioral realism is a powerful predictor of perceived social presence. These positive effects are most consistently found when the avatar's or agent's behavior indicates awareness of their communication partner's presence (e.g., mutual gaze, nodding at appropriate times, blushing). For example, von der Pütten et al. (2010) found that participants felt higher levels of social presence when they interacted with a computerized agent (Rapport Agent) that displayed appropriate feedback behavior by nodding its head compared to one that did not. Similarly, Pan et al. (2008) found that participants felt the highest level of social presence when a virtual agent blushed strongly (whole-face blush) after making a mistake during a presentation. Participants also felt higher levels of social presence when their communication partner maintained longer mutual eye contact with them compared to when he or she did not (Bente et al., 2008, Study 1); when the duration of the mutual eye gaze was too long (which is behaviorally unrealistic), however, participants responded negatively (Bente et al., 2008, Study 2). The significance of behavioral realism in fostering a sense of social presence may also explain why previous studies failed to find a positive association between the use immersive avatar-mediated VR systems and social presence. More specifically, the lack of significant results may have been due to the fairly limited level of behavioral realism afforded by older platforms.

In contrast to the relatively consistent effects of behavioral realism on social presence, studies on the impact of photographic and anthropomorphic realism reveal mixed results. While some studies show an increase in social presence when the visual representation is more photographically or anthropomorphically realistic (e.g., Kang and Watt, 2013), others report no differences (e.g., Bailenson et al., 2001; Bente et al., 2008) or even a reduction in social presence (e.g., Nowak and Biocca, 2003). The inconsistency in these results may be explained by three factors. First, photographic realism may simply not be the most crucial component of social presence. As Blascovich et al. (2002) and Nass et al. (1994) argue, the appearance of the visual representation might simply be less important than behavioral social cues. Second, the social presence questionnaires used may not have been sensitive enough to capture the subtle differences caused by variations in the appearance of the virtual human. Finally, these inconsistent effects may be explained by the varying levels of behavioral realism in each study. Studies that manipulate both the appearance *and* behavior of the visual representation show strong support for consistency effects (Garau et al., 2003; Bailenson et al., 2005). That is, participants feel greater social presence when the level of behavioral realism is consistent with the level of photographic realism. Garau et al. (2003) found, for example, that while increasing the level of photographic realism did *not* have a main effect on social presence, participants felt higher levels of social presence when they interacted with an avatar high in photographic realism compared to one low in photographic realism when the avatar displayed realistic eye gaze behavior (i.e., high behavioral realism). The opposite effect was found for avatars low in behavioral realism. In a separate study, Bailenson et al. (2005) also noted that the consistency between behavioral and photographic realism positively predicts social presence.

To summarize, the current literature offers evidence that (1) the presence of a visual representation and (2) a more behaviorally realistic visual representation enhance social presence. In contrast, while both photographic and anthropomorphic realism can enhance perceptions of social presence, this effect appears to be contingent on certain boundary conditions, including consistency with the level of behavioral realism.

#### Interactivity

While real-time virtual communication between actual people is usually characterized by high levels of interactivity, the level of interactivity afforded by a computerized agent can vary. As such, studies that explored the impact of interactivity on social presence generally looked into how an agent's interactivity influences social presence. Considering that social presence is dependent on how strongly one feels that he or she is talking with an intelligent being that is aware of his or her presence (Biocca, 1997), it is unsurprising that the extant research, albeit with some boundary conditions, offers robust evidence that interactivity is positively associated with social presence. In their study on social agents, for example, Skalski and Tamborini (2007) invited participants to listen to a health message on blood pressure. They found that participants who were given the opportunity to interact with the agent by letting it know the order in which they wished to receive the health information felt higher levels of social presence compared to participants who did not have this opportunity. Fortin and Dholakia (2005) similarly found positive effects of interactivity, although their results were qualified by participants' need for cognition (NFC); participants high in NFC showed a linear increase in social presence as the level of interactivity increased, while those low in NFC exhibited a ceiling effect wherein social presence increased between low and medium levels of interactivity, but plateaued for medium and high levels of interactivity.

#### Haptic feedback

Due to the significance of touch in physical interactions, a lot of effort has been—and continues to be—made to introduce interpersonal touch through haptic devices in virtual environments. The current review identified haptic feedback as one of the most commonly studied immersive qualities that influence social presence, apart from visual representation and interactivity. With the exception of one study (Sallnäs et al., 2000), all of the 10 studies that we identified found a positive relationship between haptic feedback and perceptions of social presence (Table 1). For example, participants felt higher levels of social presence when they received haptic feedback as they lifted a (virtual) box with a partner compared to when such feedback was not available (Kim et al., 2004). One thing to note is that, as Table 2 shows, most of the studies on haptic feedback reviewed in the present paper required participants to jointly manipulate an object (e.g., move blocks together, play air hockey). As such, the tasks themselves may have been biased to amplify the positive effects of haptic feedback compared to tasks that require less “manual” collaboration.

#### Depth cues (stereoscopy and motion parallax)

Stereoscopic displays create the illusion of depth by providing slightly different images to each eye. Motion parallax is a monocular depth cue wherein people perceive objects closer to them to be moving at a faster rate than objects a further distance. The studies that were identified in the present paper (Mühlbach et al., 1995; Takatalo et al., 2011; Kim et al., 2012; Ahn et al., 2014) suggest that the inclusion of depth cues increase social presence. In one study, for example, college freshmen viewed a virtual character (computerized agent) as it gave a 5-min news presentation about the school that they would be attending in either a stereoscopic or monoscopic display (Ahn et al., 2014). The researchers found that stereoscopy significantly increased perceptions of being together with the virtual character. Mühlbach et al. (1995) similarly found that participants felt greater social presence when they engaged in a video conferencing session using a stereoscopic display compared to a monoscopic one. Although the researchers of this study used “telepresence” to describe their outcome variable, the measures that they used (“It was as if we were all in the same room” and “It was like a real face-to-face meeting”) reflected social presence, rather than telepresence. While these studies point to a positive relationship between stereoscopy and social presence, more research is needed to support this hypothesis.

#### Audio quality

Research that investigated the impact of audio quality on social presence generally focused on how altering the number of sound channels influences perceptions of social presence. Surprisingly, we were unable to identify studies that addressed the impact of audio disturbances such as noise or dropout on social presence. While we only found three studies that manipulated audio quality, all of these studies found that improving audio quality leads to an increased sense of social presence. For example, Skalski and Whitbred (2010) conducted a study wherein participants were assigned to play a first-person shooter video game with either a 6-channel (Dolby 5.1) or 2-channel (Dolby Stereo) sound system. They found that the high audio-quality participants felt higher levels of social richness (i.e., social presence) than their low audio-quality counterparts. The authors also manipulated image quality, but no interaction effects were found between image and audio quality. In perhaps one of the earliest studies of social presence, Christie (1974) conducted a study wherein 36 businessmen discussed an important business topic in groups of six, and found that participants reported higher levels of social presence for the multi-speaker phone system than for the standard or high-fidelity speakerphone.

#### Display

A small number of studies also manipulated features of the display itself, namely image definition and display size, to examine their influence on social presence. The results of these studies yield mixed results. While two studies (Bracken, 2005; Ahn et al., 2014) found that more immersive displays (i.e., higher definition, larger screen size) led to higher social presence, two others (Skalski and Whitbred, 2010; James et al., 2011) were unable to find a significant effect of display on social presence. As such, more research is needed to understand when and how display qualities influence social presence.

### Contextual properties and social presence

As mentioned above, recent studies have begun to expand research on the predictors of social presence from immersive qualities to contextual and individual properties. This shift in the landscape may, in part, be attributed to the fact that social presence is a subjective experience that is influenced by both the perceived physical and psychological distance between the interactants, not solely the technological qualities of a medium. As such, both contextual and individual factors that contribute to how familiar or close a virtual human feels may have an influence on social presence above and beyond immersion. The following sections will describe antecedents of social presence that are not associated with objective immersive qualities, but contextual and individual qualities that impact one's subjective perceptions of being together with another person.

#### Application of social psychology: personality/traits of virtual human

Multiple studies have applied well-established findings from social psychology for positive interpersonal evaluations (e.g., similarity attraction, social penetration theory, social identity theory, preference for consistency, etc.) to technology-mediated contexts to explore their relevance in interpersonal perceptions (e.g., Reeves and Nass, 1996; Jin, 2012; Verhagen et al., 2014). This line of research has found that most interpersonal dynamics that can be found in FtF contexts can be replicated in virtual environments with both agents and avatars. For example, Qiu and Benbasat (2010) found that participants were more likely to feel higher levels of social presence when they interacted with a virtual product recommendation agent whose appearance matched their ethnicity than one that did not, replicating findings based on social identity theory (Tajfel, 1979). In another study (Kang and Gratch, 2014), participants perceived more social presence when their virtual counselor (computerized agent) disclosed more personal information about itself, which offers support for Altman and Taylor's (1973) self-disclosure theory. Similarly, participants felt higher levels of social presence when their partner's virtual representation was similar to his or her actual physical appearance (Jin, 2012), which resonates with findings regarding preference for consistency (Festinger, 1962). These studies underscore the fact that social presence is not only influenced by immersive qualities that can objectively provide richer social cues, but also by psychological processes that allow individuals to interpret the available social cues in more positive (or negative) ways.

#### Agency

Differences in agency occur depending on whether or not the virtual human is controlled by an actual human (i.e., avatar) or a computerized algorithm (i.e., agent). Studies that explore the impact of (perceived) agency on social presence generally introduce the virtual human as an actual person or a computerized character prior to the interaction. Approximately half of the studies surveyed in this paper found that people felt higher levels of social presence when the virtual human was thought to be controlled by an actual person rather than by a computer program. For example, participants felt greater social presence when they believed that the Rapport Agent they were interacting with was a real person compared to when they thought it was an artificial intelligence (Appel et al., 2012). These results are in line with Blascovich et al. (2002) model of social influence, which posits that avatars require a lower threshold of realism than agents to yield social influence. While they did not explore the impact of agency on social presence using a questionnaire, another study showed that participants showed higher physiological arousal while playing a computer game when they thought their opponent was an avatar compared to when they thought it was an agent (Lim and Reeves, 2010). These findings echo a meta-analysis conducted by Fox et al. (2015) that found that avatars generally elicit greater social influence than agents.

The remaining half of the studies, however, suggests that participants perceive similar levels of social presence for both agents and avatars (Nowak and Biocca, 2003; von der Pütten et al., 2010; Dalzel-Job, 2014, Study 2; Kothgassner et al., 2014, 2017; Felnhofer et al., 2018). Considering the fact that the majority of the studies published prior to 2010 found a positive relationship between agency and social presence (4 out of 5 studies), while only a small number of the studies published after 2010 did (2 out of 7 studies), it is possible that users have started to develop different expectations regarding how an avatar (vs. agent) should behave and/or look in virtual environments, and that deviations from these media expectations can lead to less social presence or doubt of the veridicality of the experimental manipulation, regardless of purported agency.

#### Physical proximity

The present paper also identified five studies that explored the impact of absolute physical distance between interactants on feelings of social presence (e.g., Gajadhar et al., 2008; Croes et al., 2016; Järvelä et al., 2016). These studies consistently show a positive relationship between physical proximity and perceptions of social presence. To explore the impact of physical proximity on social presence, this line of research compared the social presence of participants who had completed an activity in the same room to those who had completed the same activity in different rooms. Of note is that participants who were in the same room were often able to see each other during the interaction, while those that were placed in separate locations remained visually anonymous. As such, it is difficult to determine if the purported effects of physical proximity were driven by physical closeness, visual (non)anonymity, or both. Only two of these studies (Hatta and Ken-ichi, 2008; Croes et al., 2016) were able to effectively separate the effects of visual anonymity from physical co-location. Croes et al. (2016) study found that both physical co-location and visibility (non-anonymity) separately and positively predicted social presence. Hatta and Ken-ichi (2008) found an interaction between physical proximity and visibility, such that while physical closeness did lead to higher levels of social presence for visually anonymous partners, this effect did not persist when partners could see each other. In light of these findings, it is possible to conjecture that the positive association between social presence and physical proximity found in the remaining studies stemmed from a combination of physical co-location and visibility. In sum, there is cogent evidence that physical closeness with the interaction target contributes to perceived psychological distance and social presence, but it is likely that this effect will be influenced by the visibility of the virtual partner.

#### Task type

Four studies (Kim et al., 2013a; de Greef, 2014; Herrewijn and Poels, 2015; Wu et al., 2015) explored the influence of task type on perceived social presence. In one study (Kim et al., 2013a), participants either took care of or were taken care of by a robot. The researchers found that participant felt higher levels of social presence when the robot was the caregiver, compared to when they were asked to take care of the robot. In another study, participants felt lower levels of social presence when they were asked to observe their partner play a multiplayer game compared to when their partner observed them or when they played the game together with their partner (Herrewijn and Poels, 2015). While it is difficult to draw definitive conclusions from these studies due to the small sample size, they suggest that tasks that encourage self-directed attention (i.e., encourage the virtual human to focus on the participant) may increase social presence. Just as nonverbal cues that implied the virtual human's awareness of the participant increased social presence (e.g., Bente et al., 2008; Shahid et al., 2012), people may feel higher levels of social presence when the given task requires the virtual human to pay attention to and accommodate their behavior. More details about the tasks are given in Table 2.

#### Social cues about the presence of others

More recent studies (Choi and Kwak, 2017, Study 2; Lee and Nass, 2004; Lee et al., 2005; Kim and Sundar, 2014; Daher et al., 2016; Kim, 2016; Robb et al., 2016) have examined how the number of people or the mere presence of another person who is aware of the virtual environment (i.e., social cues) impacts feelings of social presence. In general, these studies show that seeing other people who share or interact with the same virtual environment as the user increases social presence. For example, Daher et al. (2016) found that being exposed to a conversation between a virtual human and a real person prior to the study increased feelings of social presence for the participant after interacting with the same virtual human. Choi and Kwak (2017, Study 2) found that participants felt a stronger sense of social presence when they were communicating with multiple remote partners via a telepresence robot compared to a single remote partner. These results are in line with the findings of Kim (2016) and Lee and Nass (2004), who also found that multiple virtual communicators increase feelings of social presence. In contrast to these findings, Robb et al. (2016) found that having a human teammate did not appear to increase the perceived social presence of a virtual medical practitioner. Overall, however, the majority of the research suggests that being in a context wherein individuals are exposed to cues that indicate a social context (e.g., conversation, partner, group, etc.) can lead to heightened levels of social presence. Considering the non-significant findings of Robb et al. (2016) and the relatively small number of studies, however, more research is needed to conclusively understand the implications of co-present others on social presence.

#### Identity cues

Finally, studies have also explored the provision of identity cues (e.g., name, portrait picture) as a contextual factor that influences social presence, and found that increasing the number of identity cues enhances feelings of social presence (Li et al., 2015; Feng et al., 2016; Choi and Kwak, 2017; Schumann et al., 2017). Given the fact that social presence is contingent on the extent to which an individual feels that he or she is in the presence of a “real person,” it is natural that providing participants with cues that offer insight into the “true” identity of their virtual partner(s) enhances social presence.

### Individual differences and social presence

#### Demographic characteristics: gender and age

As can be noted in Table 1, two of the most commonly examined individual differences in relation to social presence are the gender and age of the user. Most of the studies that explored the relationship between users' demographic variables and social presence did not specifically focus on these demographic variables, but included them as covariates or control variables in their analyses. In terms of gender, the majority of the surveyed studies found that females experience higher levels of social presence compared to males (e.g., Giannopoulos et al., 2008; Johnson, 2011). Age, in contrast, does not appear to have a strong association with social presence. The age range of the seven studies that explored the relationship between age and social presence are as follows: Cho et al. (2015): 21–44, Felnhofer et al. (2014): range not reported (*M* = 23.34, *SD* = 2.73), Hauber et al. (2005): 19–63, Kim et al. (2004): not reported, Lim and Richardson (2016): 24–58, Richardson and Swan (2003): 19–63, Siriaraya and Ang (2012): 22–80. Five of these found no significant relationship between age and social presence. However, considering the fact that the remaining two studies (Siriaraya and Ang, 2012; Felnhofer et al., 2014) both found that older participants tended to experience lower levels of social presence, it may be worth exploring if factors such as familiarity with a given technology or openness to new experiences influence perceptions of social presence.

#### Psychological traits

As can be noted in Table 1, more recent research explored the impact of psychological traits on social presence (e.g., Giannopoulos et al., 2008; Jin, 2010; Cortese and Seo, 2012; Kim et al., 2013a). These studies either looked at the impact of an individual's (1) propensity to become immersed in a virtual environment (e.g., immersive tendency, transportability) or (2) attitudes toward social interactions (e.g., communication apprehension, interdependent construal, extraversion, need to belong) on social presence. These studies showed that people who have stronger immersive tendencies are also more likely to experience stronger social presence. For instance, (Kim et al., 2013a) found that participants who were higher in immersive tendency were more likely to feel stronger social presence when interacting with a social robot. More interestingly, studies also found that individuals who value or enjoy social interactions experience higher levels of social presence. Jin (2010), for example, found that individuals who had interdependent self-construals experienced stronger social presence. There are two non-exclusive explanations for these findings; first, individuals who have positive attitudes toward social interactions may have a stronger desire to feel social presence and thus try harder to gratify this motivation during a virtual interaction. Second, people who are less socially oriented may lack the ability to adequately attend to the social information at hand (Cortese and Seo, 2012), and consequently experience lower levels of social presence than their more socially oriented counterparts even with the same amount of social cues. Overall, these studies highlight the importance of considering individual differences when examining features in a virtual environment that might influence social presence.

## Discussion

**Figure 2:**
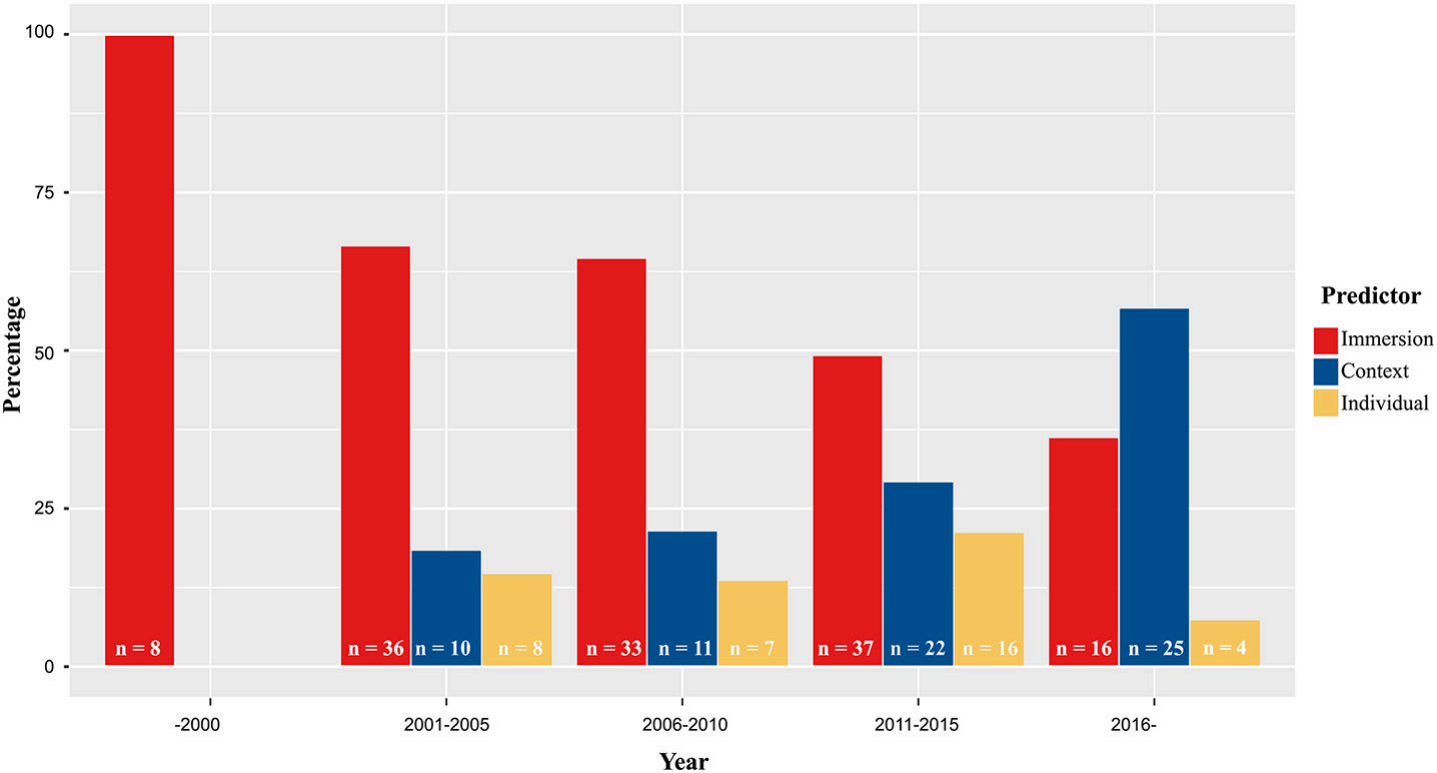
Proportion of studies that examine immersive, contextual, and individual predictors of social presence.

Thus far, the present paper defined social presence and explored the technological, contextual, and individual qualities that can influence perceptions of social presence. Overall, we found that immersion and context have a positive effect on social presence, although there do appear to be ceiling effects and boundary conditions. While demographic information, and psychological traits associated with positive attitudes toward social interactions also tended to increase participants' feelings of social presence, the effects of demographic characteristics were less conclusive. Although we interpreted null findings to indicate the absence of a significant effect, it is important to note that several of the studies were conducted on a small number of participants (see Table 3). As such, some of the non-significant results can also be interpreted as inconclusive findings, and thus merit further research. As mentioned before, while earlier studies on the predictors of social presence focused primarily on the impact of immersive features, a growing number of researchers have begun to consider contextual and individual features as factors that can increase or decrease feelings of social presence (Figure 2).

One caveat to the present review is that social presence was operationalized in a number of different ways depending on the study (see Table 2). Considering the fact that questionnaire wording can influence responses (Borgers et al., 2004), it is possible that the use of different measures may account, at least in part, for why the same feature predicted social presence in some cases, but not in others. As social presence is often measured in different contexts (e.g., human-agent interaction, human-human interaction, etc.), some diversity in measures is inevitable (Biocca et al., 2003). However, more effort is needed to build a “foundation for theory and measure of social presence with greater explanatory and predictive power” (Biocca et al., 2003, p. 474).

**Figure 3:**
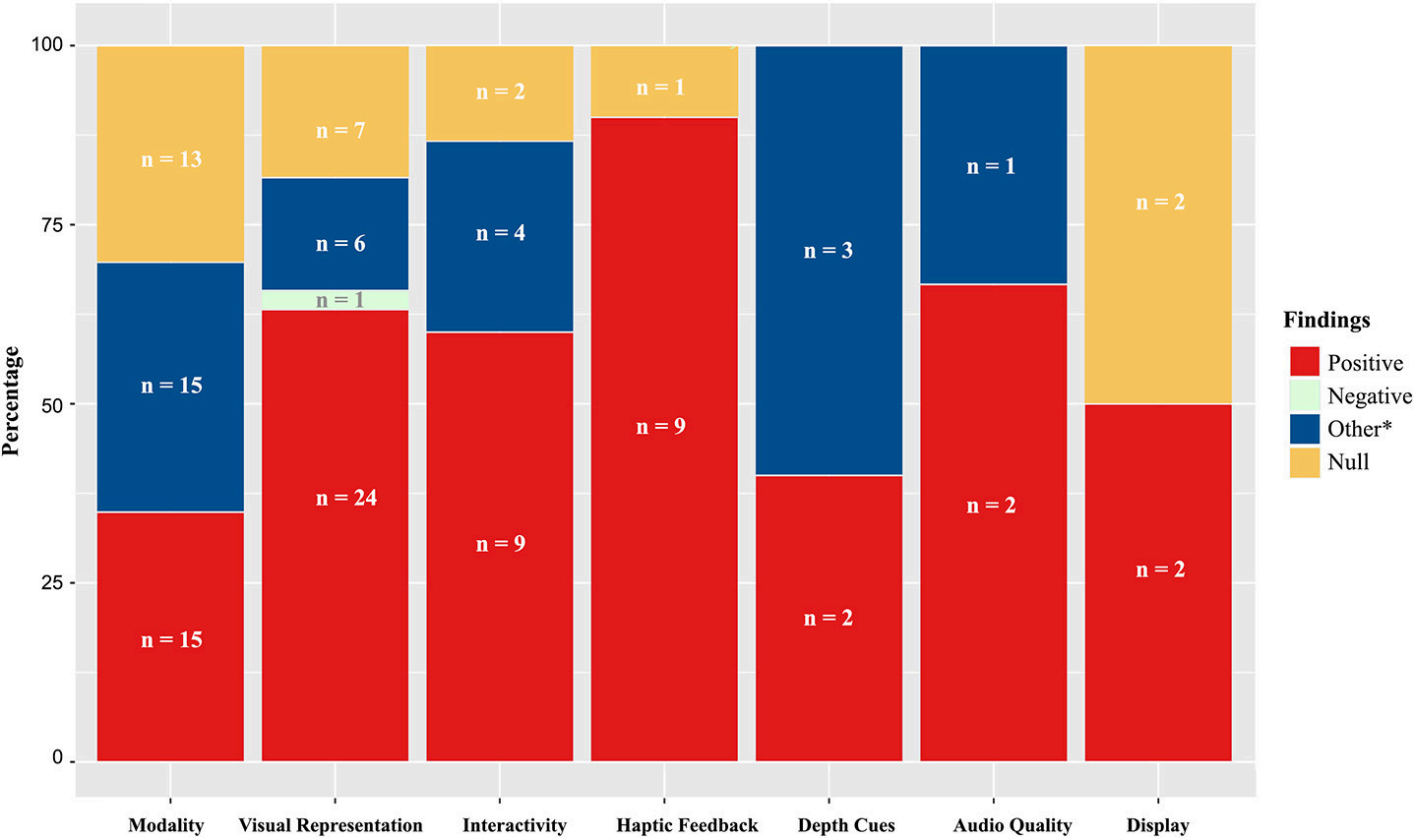
Effects of immersive features on social presence. *Other refers to moderated or non-linear results.

**Figure 4:**
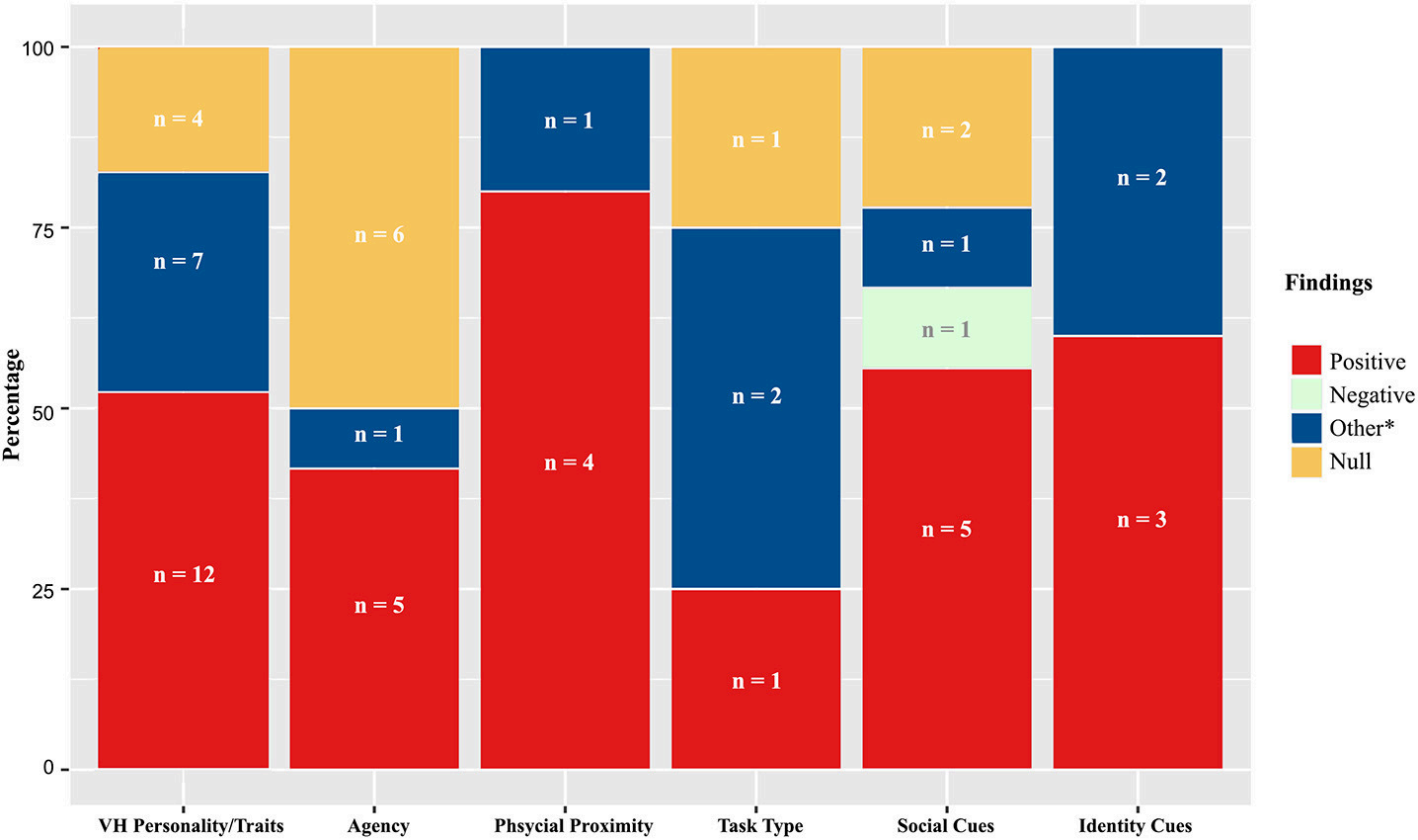
Effects of contextual features on social presence. *Other refers to moderated or non-linear results.

**Figure 5:**
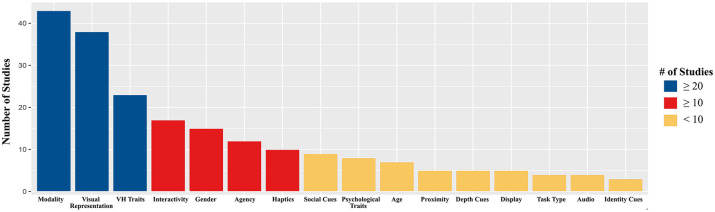
Number of studies that explore each antecedent of social presence.

While the variability of sample size per predictor necessitates caution in interpreting our results, we found that depth cues, audio quality, haptic feedback, and interactivity often had positive effects on social presence (Figure 3). In contrast, there influence of general modality, visual representation, and display were somewhat weaker. Among contextual factors, physical proximity, identity cues, and the personality/traits of the virtual human were often significant predictors of social presence. Somewhat surprisingly, the effects of agency were less conclusive (Figure 4). In terms of demographic factors, neither age nor gender appeared to have a clear effect on social presence. In contrast, certain psychological traits (e.g., transportability, extraversion, need to belong) tended to predict social presence. However, as much of the available research focuses on a select number of predictors such as general modality, visual representations, and personality/traits of the virtual other, more studies are needed before we can draw concrete conclusions about the impact of certain features (Figure 5).

One interesting point to note is that the majority of the studies identified in this paper frame social presence as an “absolute good.” Social presence is often used to assess how “successful” a given communication system is at emulating the gold-standard of FtF communication (e.g., Biocca et al., 2001; Hauber et al., 2005). In addition, researchers frequently hypothesize that increasing the salience of the mediated communication partner will naturally lead to more positive social outcomes (e.g., Fogg and Tseng, 1999; Hassanein and Head, 2007). While there is a wealth of research that supports this claim, this approach misleads researchers to neglect the fact that social presence may not always yield positive outcomes. This is an important issue to consider when designing communication systems; more social presence may not always be better (Allmendinger, 2010). Therefore, it is necessary to consider the characteristics of the communicator as well as the context in order to leverage the unique possibility of offering varying levels of social presence within virtual environments.

Attempts to increase social presence may lead to negative communication outcomes when the communicator is a person who feels discomfort during social interactions. Individuals who have high social anxiety or communication apprehension are generally uncomfortable in the presence of people. As such, these individuals prefer to withdraw from social situations and stay in the background, rather than engaging in the conversation (Allmendinger, 2010; Cortese and Seo, 2012). Consequently, they may feel more comfortable when the social presence of their communication partners is low, rather than when it is high. The fact that individuals who do not value or enjoy social interactions (e.g., shy, high communication apprehension, weaker need to belong, etc.) feel less social presence than their more social counterparts (Giannopoulos et al., 2008; Jin, 2010; Cortese and Seo, 2012; Kim et al., 2013a) offers some empirical evidence that socially withdrawn individuals may be less motivated to attend to social cues that enhance social presence. Directly germane to this hypothesis, studies (Joinson, 2004; Hertel et al., 2008; Hammick and Lee, 2014) consistently show that less socially oriented individuals prefer interacting through a medium that is considered to be “leaner” (e.g., text-based CMC), while more socially oriented individuals prefer to interact via a “richer” modality (e.g., FtF). Similarly, Poeschl (2017) found that perceiving the virtual audience to be more socially present tended to lead to a worse speech-giving performance.

The desirability of social presence may also differ depending on the interaction context. Studies suggest that higher levels of social presence are more beneficial in equivocal contexts wherein there is no “correct” outcome, such as negotiations (Daft and Lengel, 1986; Garau et al., 2003). In contrast, it is possible that people will prefer lower levels of social presence when they are feeling more vulnerable; the success of text-based counseling and support systems (Dinakar et al., 2015) lends some support to this conjecture. Taken together, these studies suggest that attempting to increase social presence may not have uniformly positive results; rather, special attention should be paid to the communication preferences and goals of the interactants.

In addition to individual traits, social presence may have differential communication outcomes depending on one's attitude toward his or her communication partner. That is, while increasing the salience of a neutral or likable communication partner may increase positive social outcomes, enhancing the social presence of a disliked communication partner might lead to less desirable results. As Lee and Shin (2012) argue, increased social presence of a disliked target can escalate the negative thoughts associated with him or her, which may in turn amplify prior attitudes toward the target. The fact that gamers felt more hostile and were more verbally aggressive toward their opponent when they experienced stronger levels of presence during a violent game (Nowak et al., 2008) offers some support to this hypothesis. Lee and Shin (2012) also found that while higher social presence of a high-profile politician led to stronger agreement with his opinions when participants liked him, this was not the case when participants did not have positive pre-dispositions toward him.

Considering these boundary conditions of the benefits of increased social presence, researchers should focus not only on the predictors of social presence, but also the interpersonal outcomes of enhanced social presence. Doing so will offer a more holistic view of social presence that will allow for a better understanding of when it is (and is not) desirable for a virtual environment to adopt immersive and contextual qualities that will increase social presence.

## Limitations

There are several limitations in the current study. First the research that was reviewed often used different measures of social presence, which limits their comparability. It is thus important for researchers to note the different measures used (available in Table 2), to contextualize the findings of each study that was reviewed. We chose not to conduct a quantitative meta-analysis due to the variability of measures, as we believed this approach would lead to the exclusion of a number of important studies.

In addition, we did not assess the quality of each study that was included in this review; rather, we assumed that the findings of each study were true and correct. However, we included the publication outlet, number of citations, and the impact factor of the publication outlet (when available) in addition to the number of participants in Table 3. While these factors are not definitive criteria in determining the quality of a study, we hope that they will help readers better understand the nature of each study.

Another limitation of the present study is that we were unable to include concepts that share theoretical similarities with social presence. While this decision was made to achieve a higher level of internal validity, it led to the exclusion of research on important concepts, one of which is plausibility illusion (Slater, 2009; Slater et al., 2010). Plausibility illusion research significantly contributes to understanding when and how people respond to virtual others as “real” people, as it encapsulates the extent to which one feels as if the depicted events are actually occurring. In contrast to “the sense of being there” (i.e., place illusion), which tends to be contingent on the technological characteristics of the environment, Plausibility illusion concerns the *credibility* of a scenario, and thus is not dependent on the sensory capabilities of a virtual environment (Slater and Sanchez-Vives, 2016; Gonzalez-Franco and Peck, 2018). Although plausibility illusion is not identical to the concept of social presence (see Methods section), it can inform social presence researchers on why higher levels of immersion do not universally lead to higher levels of social presence. More importantly, plausibility illusion research can offer insight into when and how non-technological factors (e.g., mimicry, task type, etc.) influence the believability of the virtual human. In one study on bystander effects in a virtual bar, for example, participants reported contextual factors (e.g., appearance of bar, responsiveness of other characters, believability of dialogue with victim) as issues that brought them out of the virtual experience (“breaks in presence” Slater and Steed, 2000; Slater et al., 2013). Researchers have also explored the impact of the personality of the virtual human (Pan et al., 2015), level of coherence to the user's expectations (Skarbez et al., 2017), and the physicality of the virtual human (Chuah et al., 2013) on plausibility illusion.

Our study also did not explore the how the *actual* agency of the target influences social presence; while we did review studies that examined how agency affects social presence, they addressed perceived, rather than actual agency. While manipulating perceived agency does maximize internal validity, it reduces some of the external validity, given that avatars and agents are likely to behave differently outside of the laboratory. Although this is beyond the scope of the present study, we have included a column in Table 2 that notes whether or not the evaluation target in each study was an actual person or a computer algorithm.

Finally, the present study did not address potential moderators that could influence the impact of each feature on social presence. As we discussed above, both individual and contextual factors may moderate the findings of our systematic review. Future studies would benefit from exploring potential moderators and their relative effects.

## Conclusion and future directions

Despite its potential drawbacks, social presence is a critical experience within networked environments. While increased social presence may not always lead to positive results, multiple studies show that the vivid perceptions of another person often lead to greater enjoyment and social influence in neutral and positive contexts (e.g., Fogg and Tseng, 1999; Hassanein and Head, 2007). Hence, a considerable amount of scholarly efforts have been made to identify factors that increase feelings of social presence, as we have found in the present paper. By reviewing these studies, we were able to identify immersive, contextual, and individual qualities that impact perceptions of social presence.

It is important to note, however, that due to the period during which they were conducted, many of these studies employed limited technology, and thus do not address the implications of recent technological advancements. For example, many VR systems now offer inverse kinematics, the prediction of joint movements based on the position(s) of a limited set of trackers. Considering the fact that both gesture and posture have a significant influence on person perception in FtF contexts (Riggio and Friedman, 1986), it is possible that this added layer of technology in CMC will impact experiences of social presence. However, this possibility has not been fully explored within the current social presence literature. Similarly, studies have also failed to explore the implications of rendering expressions that are driven by facial motion tracking data, another recent technological development. These research questions are important both from a theoretical and applied standpoint. From a theoretical point of view, these questions offer insight into the social cues that are necessary to induce feelings of a “social being,” or what it means for a virtual entity to “appear human.” In addition, these questions allow us to explore how immersive VR systems that support unprecedentedly high levels of behavioral realism influence social presence. From an applied point of view, this research will allow system designers to understand how to allocate resources when developing networking systems.

Future studies on social presence would also benefit from considering plausibility illusion research when formulating hypotheses. In addition, more empirical research is needed on the theoretical similarities and differences between social presence and plausibility illusion. For example, while there is evidence that the personality of the virtual human (e.g., friendliness, empathy, etc.) influences social presence, it is less probable that these features will influence plausibility illusion, or how believable they find the virtual human's behavior to be. Lending some support to this conjecture, Pan et al. (2015) found that the shyness of a virtual human did not influence perceptions of plausibility illusion. In contrast, it is reasonable to conjecture that behavioral realism will positively influence both social presence and plausibility illusion. This line of research can aid in creating a more cohesive theoretical framework for presence and its components, fostering fruitful intra- and inter-disciplinary discussions between VR researchers.

In addition, future studies should offer a more holistic view of social presence by considering the different dimensions that impact social presence. Just as studies found that increasing the behavioral realism of a virtual human that had low photographic realism did not lead to increased social presence (Garau et al., 2003), it would be beneficial to consider boundary conditions (e.g., contextual, individual) of the findings available in the current literature. One understudied, but important, boundary condition is the relationship between the conversation partners. Given that technological features such as audio delays differentially influence communication outcomes depending on the relationship between the partners (Koudenburg et al., 2014), this avenue of research may help researchers and practitioners understand how to design social VR systems when individuals are already acquainted with each other. Furthermore, considering that multiple studies reviewed in this paper show that increasing immersive qualities does not linearly increase social presence (e.g., Moreno and Mayer, 2004; Sallnäs, 2005; Homer et al., 2008), it would be critical to understand if, and if so when, there is a ceiling effect of immersion on social presence.

Lanier (2014) noted that a good VR system should be “good enough to fool you, to engage your whole body, to include others as avatars with you in there, to be usable in the long term, and giving you enough to do to outlast the first few demos” (p. xiii). However, he cautions that such high quality VR is still only available at a limited number of places. With the popularization of VR at the horizon, it is essential for both academic and industrial researchers to increase their understanding of what helps create the sense of being there with other people in this space of “consensual hallucination” (Gibson, 1984, p. 51).

## Author contributions

CO, JB, and GW contributed to the conception and design of the study. CO organized and reviewed the database of social presence studies. CO wrote the first draft of the manuscript. CO, JB, and GW contributed to the manuscript revision, read and approved the submitted version.

### Conflict of interest statement

The authors declare that the research was conducted in the absence of any commercial or financial relationships that could be construed as a potential conflict of interest.
